# NMPA-approved traditional Chinese medicine-Pingwei Pill: new indication for colistin recovery against MCR-positive bacteria infection

**DOI:** 10.1186/s13020-021-00518-y

**Published:** 2021-10-18

**Authors:** Qiushuang Sheng, Runbao Du, Cunhui Ma, Yonglin Zhou, Xue Shen, Xiaoning Hou, Lei Xu, Li Li, Xuming Deng, Jianfeng Wang

**Affiliations:** Key Laboratory of Zoonosis Research, Ministry of Education, Institute of Zoonosis, College of Veterinary Medicine, Jilin University, Changchun, China

**Keywords:** Synergistic effect, Pingwei Pill, Colistin, MCR, Anti-infection, Pathway verification, Network pharmacology

## Abstract

**Background:**

The wide spread of plasmid-mediated colistin resistance by mobile colistin resistance (MCR) in Enterobacteriaceae severely limits the clinical application of colistin as a last-line drug against bacterial infection. The identification of colistin potentiator from natural plants or their compound preparation as antibiotic adjuncts is a new promising strategy to meet this challenge.

**Methods:**

Herein, the synergistic activity, as well as the potential mechanism, of Pingwei pill plus antibiotics against MCR-positive Gram-negative pathogens was examined using checkerboard assay, time-killing curves, combined disk test, western blot assay, and microscope analysis. Additionally, the *Salmonella* sp. HYM2 infection models of mouse and chick were employed to examine the in vivo efficacy of Pingwei pill in combination with colistin against bacteria infection. Finally, network pharmacology and molecular docking assay were used to predicate other actions of Pingwei pill for *Salmonella* infection.

**Results:**

Our results revealed that Pingwei Pill synergistically potentiated the antibacterial activity of colistin against MCR-1-positive bacteria by accelerating the damage and permeability of the bacterial outer membrane with an FIC (Fractional Inhibitory Concentration) index less than 0.5. The treatment of Pingwei Pill neither inhibited bacterial growth nor affected MCR production. Notably, Pingwei Pill in combination with colistin significantly prolonged the median survival in mouse and chick models of infection using the *Salmonella* sp. strain HYM2, decreased bacteria burden and organ index of infected animal, alleviated pathological damage of cecum, which suggest that Pingwei Pill recovered the therapeutic performance of colistin for MCR-1- positive *Salmonella* infection in mice and the naturally infected host chick. Pharmacological network topological analysis, molecular docking, bacterial adhesion, and invasion pathway verification assays were performed to identify the other molecular mechanisms of Pingwei Pill as a colistin potentiator against Gram-negative bacteria infection.

**Conclusion:**

Taken together, NMPA (National Medical Products Administration)-approved Pingwei Pill is a promising adjuvant with colistin for MCR-positive bacterial infection with a shortened R&D (research and development) cycle and affordable R&D cost and risk.

**Supplementary Information:**

The online version contains supplementary material available at 10.1186/s13020-021-00518-y.

## Background

Conventional regimens to address bacterial infectious disease have induced serious bacterial resistance worldwide [1–4]. This problem is more complicated by the emergence of multidrug resistance (MDR) [5, 6]. Enterobacteriaceae is the main pathogen of Gram-negative bacteria, which causes severe threats and economic losses to public health and animal husbandry [7]. Colistin was considered the final choice for MDR bacteria. However, the therapeutic effect of colistin was threatened by the appearance and spread of mobile colistin resistance, which is a phosphoethanolamine transferase encoded by *mcr* and was identified as a source of acquired resistance to colistin. The colistin resistance gene *mcr* was identified in more than 50 countries worldwide [8–14]. Drug-resistant bacteria carrying MCR-1 were detected and reported ubiquitously in rivers and soil in natural environments, especially animal food (pigs, cattle, and poultry). As the expansion of *mcr* gene family members, variant genes from *mcr*-1 to *mcr*-10 were reported, which suggests that the *mcr* gene has spread around the world and is constantly evolving [15]. The NCBI database revealed that MCR-1-positive plasmids have spread worldwide in different species of Enterobacteriaceae, including *Salmonella*, *Klebsiella*, *Enterobacter* and *Shigella.* All these findings are a wake-up call to the entire world in the fight against bacterial resistance, especially MCR.

Recent research has started to identify inhibitors against MCR. A recent study [16], designed small molecule compounds according to MCR structure and identified 1-phenyl-2-(phenylamino) ethenone derivatives as effective MCR inhibitors. Monomeric compounds extracted from natural plants also have these inhibitory effects [17–20]. However, neither small molecule compounds nor Chinese herb monomers were immediately given to animals or humans. The rate of new drug development cycles was far from following the resistance of bacteria. The high research costs and unpredictable risks limit enthusiasm for drug discovery. Therefore, the identification of novel agents from conventional drugs as adjuncts with colistin to fight MDR bacterial infection via the targeting of MCR is a priority.

Traditional Chinese medicine (TCM) is widely and effectively used for the treatment of infection by various pathogens, including bacteria, viruses, and parasites [21, 22]. TCM also plays an irreplaceable role in the treatment of bacterial infection [23, 24]. Although ideal performance exists, the mechanisms of TCM against infection by various pathogens, especially bacteria, are not clear. Based on the MCR inhibitor screening platform, an NMPA-approved TCM was considered an effective adjunct with colistin against MDR bacteria. The advantages of the approved drugs for new clinical use were timesaving, low cost, safety, reliability, and immediate use. Fortunately, NMPA-approved TCM Pingwei Pills were identified as potential MCR-1 inhibitors and showed significant synergistic effects in combination with colistin against various *mcr*-1-positive bacteria in vitro and in vivo. Molecular docking results in this type of a synergistic effect. Together, this work successfully elucidated the inhibitory effects of Pingwei Pill against MCR-1. Network pharmacology analysis was used to characterize the potential mechanism. Focal adhesion, adherent junctions and bacterial invasion of the epithelial cell pathway were enriched by the main targets of the synergistic effect between Pingwei Pill and colistin. Pingwei Pill may represent an ideal adjunct agent with colistin against bacterial infection, which paves the way for the development of NMPA-approved TCMs as agents against infections of drug-resistant bacteria.

## Methods and materials

### Bacteria and reagents

*Escherichia coli* ATCC 25, was purchased from American Type Culture Collection (ATCC). Human clinical MCR-1-producing isolates *K. pneumoniae* ZJ02, *E. coli* ZJ40, and *E. coli* ZJ478 were collected in our previous study [7]. *E. coli* DH5α (pUC19-*mcr*-1) [20], which carries an *mcr-1* gene originating from *K. pneumoniae* ZJ05, *Salmonella* sp. strain HYM2, *Salmonella* sp. strain ZZW20 and *Salmonella* sp. strain 15E464 were provided by Professor Jian Sun [25] in South China Agricultural University. Pingwei Pill was obtained from Bei Jing Tong Ren Tang Group (Beijing, China). Colistin sulfate was purchased from the National Institute for Food and Drug Control (Beijing, China). Penicillin, Cephalothin sodium, Meropenem, Streptomycin, Kanamycin sulfate, Gentamycin, Chloramphenicol, Achromycin, Ciprofloxacin and Erythromycin were obtained from Dalian Melian Biotechnology Co., Ltd. (Dalian, China). Stock solutions of Pingwei Pill dissolved in dimethyl sulfoxide or sodium carboxymethyl cellulose solution (Sigma-Aldrich, St. Louis, MO).

### Antibacterial test

#### Checkerboard assay

The synergistic effects of antibiotics and Pingwei Pill were evaluated using checkerboard assays with two-fold serial dilutions according to the guidelines of the Clinical and Laboratory Standards Institute [26]. All compounds were diluted two-fold in LB (Luria–Bertani) broth and mixed with bacterial suspension equally in sterilized 96-well polypropylene microtiter plates. MIC values were defined as the lowest concentrations of compounds with no visible growth of bacteria between 18 and 24 h of incubation at 37 °C. The efficacies of the combinations were evaluated by calculating the fractional inhibitory concentration (FIC) index values [27] as follows: FIC index = FIC _colistin_ + FIC _Pingwei Pill_ = MIC _combination_ /MIC _colistin_ + MIC _combination_ /MIC _Pingwei Pill_. (MIC _colistin_ is the MIC of colistin alone; MIC _combination_ is the MIC of colistin in combination with Pingwei Pill; MIC _Pingwei Pill_ is the MIC of Pingwei Pill alone; Synergy is defined as an FIC index of *P* ≤ 0.5.

### Growth curves

All the tested strains were cultured in LB broth with shaking at 37 °C until the absorbance value reached 0.3 at OD_600nm_. Each bacterial suspension was equally divided into five identical conical flasks with different concentrations of Pingwei Pill (0, 0.256, 0.512, 1.024 and 2.048 mg/mL). The growth of bacteria cultured at 37 °C with shaking was evaluated by measuring the optical density at OD_600nm_ every 30 min.

### Kill-curve kinetics analysis

A time-killing curve was used to estimate the potential synergistic effect of Pingwei Pill and colistin [28]. The test strains were incubated overnight at 37 °C with shaking, diluted 1/500 in LB and incubated for 4 h (exponential phase). The bacterial cells were diluted to 5 × 10^5^ CFU/mL in LB broth treated with colistin (2 µg/mL), Pingwei Pill (1.024 mg/mL), or a combination of colistin and Pingwei Pill or DMSO (control). Following incubation at 37 °C for the indicated time points (0, 1, 3, 5, 7 and 9 h), the samples were removed, diluted appropriately, and plated on agar plates for the calculation of CFU after incubation at 37 °C overnight.

### Combined disk tests

A combined disk test was performed according to a previous report [29, 30]. The tested strain suspension with an absorbance value of 0.1 was used for LB agar plates. The indicated concentrations of Pingwei Pill (10 µL) were added to the agar plates with 10 µg colistin disks (Oxoid Ltd., Basingstoke, United Kingdom). Subsequently, the agar plates were incubated for 24 h at 37 °C before the inhibition zone diameters were observed and recorded.

### Western blot assay

The strain suspension with an absorbance value of 0.1 at OD_600nm_ was cocultured with Pingwei Pill (0, 0.256, 0.512 and 1.024 mg/mL) at 37 °C for 4 or 6 h. The sample solution was removed via centrifugation, precipitated by boiling with loading buffer, separated using SDS-PAGE and transferred onto polyvinylidene fluoride (PVDF) membranes. Following incubation with antibodies according to a previous report [9], the blots were observed using a Western blotting visualizer (Tanon 4200). The density of each band was evaluated using ImageJ software.

The strain suspensions with an absorbance value of 0.1 at OD_600nm_ were cocultured with the indicated concentrations of Pingwei Pill (0.256 mg/mL, 0.512 mg/mL, or 1.024 mg/mL) plus colistin (2 μg/mL) at 37 °C for 6 h. Then the sample solutions were performed in the same process as described above.

### Cytotoxicity assessment

The hemolytic activity of colistin with or without Pingwei Pill was evaluated. Briefly, sheep blood cells were treated with colistin (from 0 to 128 μg/mL), Pingwei Pill (from 0 to 1.024 mg/mL) or their combination for 1 h at 37 °C [31]. The absorbance of supernatants at 543 nm was measured, and hemolysis of each sample was calculated via comparison with the positive control. PBS and water were used as negative and positive controls, respectively.

Caco-2 and HeLa cells were plated on 96-well plates at 1 × 10^4^ cells/well and treated with colistin (from 0 to 128 μg/mL), Pingwei Pill (from 0 to 1.024 mg/mL) or a combination at 37 °C for 24 h. Following centrifugation, the release of LDH into the supernatants was examined using an LDH assay kit by determining the absorbance of each sample at 450 nm.

Peritoneal macrophages were obtained from C57BL /6 mice by intraperitoneal injection DifcoTM Fluid Thioglycollate Medium. After 4 days raised, the mouse was killed by cervical dislocation. Then, the peritoneal macrophage was collected by washing the abdominal region with RPMI-1640 medium with a centrifugation at 1000 rpm for 10 min. Subsequently, the peritoneal macrophage was re-suspended with RPMI-1640 medium with 10% FBS and seeded into 96-well plates at 1 × 10^4^ cells/well. The treatment process with Pingwei Pill and colistin to peritoneal macrophage was the same to Caco-2 and HeLa cells described above.

### Analysis of bacteria membrane damage

Bacterial suspensions in the logarithmic growth phase were collected, washed with phosphoric acid buffer (0.02 mmol/L, pH 7.4), resuspended to 1.0 × 10^8^ CFU/mL and equally divided into four groups. Pingwei Pill (1.024 mg/mL), colistin (2 μg/mL), the combination or solvent control was added to the strain suspensions, which were subsequently cultured on glass slides with 0.1% polylysine treatment in 24-well plates. Following incubation at 37 °C for 4–6 h, the glass slide was washed with phosphoric acid buffer three times and fixed with 2.5% glutaraldehyde for 12 h for SEM inspection (S-3400N, HITACHI).

### Bacterial viability assay

*Salmonella* sp. strain HYM2 was cultured overnight, diluted (1/50) in 10 mL fresh THY and incubated at 37 °C for 3 h until the OD_600nm_ value was 0.3. The cultures were treated with Pingwei Pill, colistin, the combination or solvent control at 37 °C for another 8 h. The bacterial pellet was collected via centrifugation at a speed of 10,000 rpm for 2 min, resuspended in 300 μL PBS and treated with 1 μL fluorescent reagent of the LIVE/DEAD® BacLight ™ Bacterial Viability Kit according to the kit’s instructions. Following incubation in the dark, 5 μL of the mixture was used for imaging with a fluorescence microscope (IX83P2ZF, Olympus).

### Serial passage assay

For each tested strain, the absorbance value of the bacterial suspension was adjusted to 0.1 at OD_600nm_ with the addition of subinhibitory concentrations of colistin, Pingwei Pill, the combination, or solvent control and further incubated for 24 h at 37 °C under shaking. The next day, the treated bacterial suspension in tubes was added appropriately to other tubes with fresh LB broth, and the OD_600nm_ value was adjusted to 0.1. Various treatments of colistin, Pingwei Pill and their combination were added to the tubes in the same as described above. The checkerboard method was performed in 96-well plates to detect changes in colistin MIC every day. Processing continued in this manner until 20 serial passages or when the passage MIC exceeded the resistance breakpoint for 2–3 consecutive passages.

### Molecular docking

The protein crystal models (5GRR, IL6, and DGKA) were obtained from the RCSB Protein Data Bank (https://www.rcsb.org/). The top 7 ingredients of Pingwei Pill were selected with OB > 30% in TCMSP, and the 2D structures of each compound were obtained from PubChem (https://pubchem.ncbi.nlm.nih.gov/). The protein receptor and small molecule ligands were docked together in Sail Vina v1.0, and the results were entered into the protein–ligand interaction profiler (PLIP) website for online analysis (https://plip-tool.biotec.tu-dresden.de/plip-web/plip/index) [32]. The visual images were exported from PyMol v2.4.0 software.

### Animal experiments

According to the animal use guidelines of Jilin University, all animal (mouse and chick) experiments were reviewed and approved by the animal experimentation ethics committee of Jilin University. For animal infection, *Salmonella* sp. strain HYM2 was grown in LB broth to mid-logarithmic phase (OD _600_ = 0.6 ~ 0.8) at 37 °C with shaking, centrifuged at 6000×*g* for 5 min, washed three times with PBS and resuspended in PBS.

Six-to-eight-week-old female BALB /c mice were purchased from Liaoning Changsheng Biotechnology Co., Ltd. and housed in groups under standard ventilated cages independently with standard food and water 3 days before the experiment. Water and food were withdrawn 4 h before intragastric administration of 10 mg streptomycin to each mouse [33]. Mice were supplied with water and food normally afterward. Water and food were withdrawn again 20 h after streptomycin treatment and 4 h before the mice were infected intragastrically with the above *Salmonella* sp. strain HYM2 suspension (1 × 10^6^ CFU/mouse for survival analysis and 1 × 10^5^ CFU/mouse for other analyses) or treated with sterile PBS as a negative control. Pingwei Pill and colistin were suspended with 0.5% CMC-Na (sodium carboxymethyl cellulose) that was used as vehicle control. Two hours later, the mice were intragastrically administered colistin (2 mg/kg), Pingwei Pill (0.8 g/kg), or Pingwei Pill (0.8 g/kg) in combination with colistin (2 mg/kg) or solution (0.5% CMC-Na) as a positive control. The dosing interval was 12 h, and the negative control mice without infection were administered PBS intragastrically on the same schedule. The survival of the mice in each group (n = 10) was determined for 1 week. The body weight of mice in each group (n = 5) was monitored for 7 days. The other mice in each group (n = 10) were sacrificed via cervical dislocation at day 3 post infection for the determination of bacterial burden in the cecum using homogenization, serial dilutions, and plating on LB agar plates under streptomycin selection. The levels of cytokines (IL-1β, IL-6, TNF-α and IFN-γ) in the supernatants of homogenized cecum tissue were detected using enzyme-linked immunosorbent assay (ELISA). For histological analysis, cecum tissue was fixed in formalin, embedded in paraffin, stained with hematoxylin and eosin (H&E), and observed under light microscopy.

A total of 100 one-day-old chicks were used for intragastric intestinal infection with the above *Salmonella* sp. strain HYM2 suspension (1 × 10^9^ CFU/chick for survival analysis and 1 × 10^7^ CFU/chick for other analyses). Then after 2 h, the chicks were intragastrically administered colistin (4 mg/kg), Pingwei Pill (1.6 g/kg), or Pingwei Pill (1.6 g/kg) in combination with colistin (4 mg/kg) or solution (0.5% CMC-Na) as a positive control. The dosing interval was 12 h, and the negative control chicks without infection were administered PBS intragastrically on the same schedule. The analyses of survival (n = 10), body weight (n = 5), bacterial burden (n = 10), histological injury (n = 10) and inflammatory response (n = 10) were performed as described for mouse infection.

### The ingredients of Pingwei Pill and potential target prediction

The ETCM (The Encyclopedia of Traditional Chinese Medicine) and TCMSP (Traditional Chinese Medicine Database and Analysis Platform) databases were used to obtain ingredients with OB (oral bioavailability) > 30% and DL (drug-likeness) > 0.18 and potential targets of Pingwei Pill (http://www.tcmip.cn/ETCM/, https://tcmsp-e.com/). Pingwei Pill related TCMs include CANG ZHU (English name: rhizoma atractylodis; Latin name: *Atractylodes lancea*), HOU PU (English name: Bark of officinal Magnolia; Latin name: *Cortex Magnoliae Officinalis*), CHEN PI (English name: Dried Tangerine Peel; Latinname: *Pericarpium Citri Reticulatae*) and GAN CAO (English name: Glycyrrhiza; Latinname: *Radix Glycyrrhizae*). The chemical-gene co-occurrences of colistin were collected from the PubChem database (https://pubchem.ncbi.nlm.nih.gov/). The disease related genes of *Salmonella* infection were obtained from GEO (Gene Expression Omnibus, https://www.ncbi.nlm.nih.Gov/geo/) and KEGG databases (Kyoto Encyclopedia of Genes and Genomes, https://www.genome.Jp/kegg/mapper/color.html). The subnetworks associated with the main targets were drawn in Cytoscape 3.7.2 software with Human Protein Reference Database Protein–Protein Interactions (hprdPPI) as the background network. The related pathways of target genes were enriched using the STRING and Metascape databases (https:// www. string. db. org/,http://metascape.org/), and a bubble diagram was obtained form OmicShare online (http://www.omicshare.com).

### Bacterial adhesions and invasion assay

HeLa cells were plated into 24‐well plates at a density of 5 × 10^5^ cells/well and incubated at 37 °C for 12 h in a CO_2_ incubator. *Salmonella *sp. cultured overnight and diluted 1:20 in LB broth, and Pingwei Pill DMSO solution was added in 0 μg/mL, 256 μg/mL, 512 μg/mL, and 1024 μg/mL, bacteria added only as positive, respectively. Then cultured for 5 h at 37 °C with 200 rpm. Afterwards, the bacteria were added to HeLa cells at an MOI of 100, colistin added at same time with final concentration was 2 μg/mL. After 20 min, the HeLa cells were washed by PBS three times. Subsequently, the cells were lysed with 0.02% triton and diluted appropriately, then plated on LB agar plates.

The steps of invasion assay, cell treatment and bacteria treatment were same to above, before washed cells, the bacteria and cells were cultured for 1 h, and the culture medium was replaced with fresh DMEM containing 100 µg/mL gentamicin for 1 h at 37 °C, 5% CO_2_. After washing three times, the cells lysed with 0.02% triton and diluted appropriately, then plated on LB agar plates.

### Data and statistical analysis

Statistical Program for Social Sciences (SPSS) version 19.0 (IBM Corp. Armonk, NY, USA) was used to analyze experimental data, which are presented as the means ± SD (n ≥ 3). Significant differences were analyzed using one-way ANOVA followed by a Dunnett t-test. *P < 0.05; **P < 0.01.

## Results

### Pingwei Pill restored the activity of colistin against *mcr*-positive bacteria

**Fig. 1:**
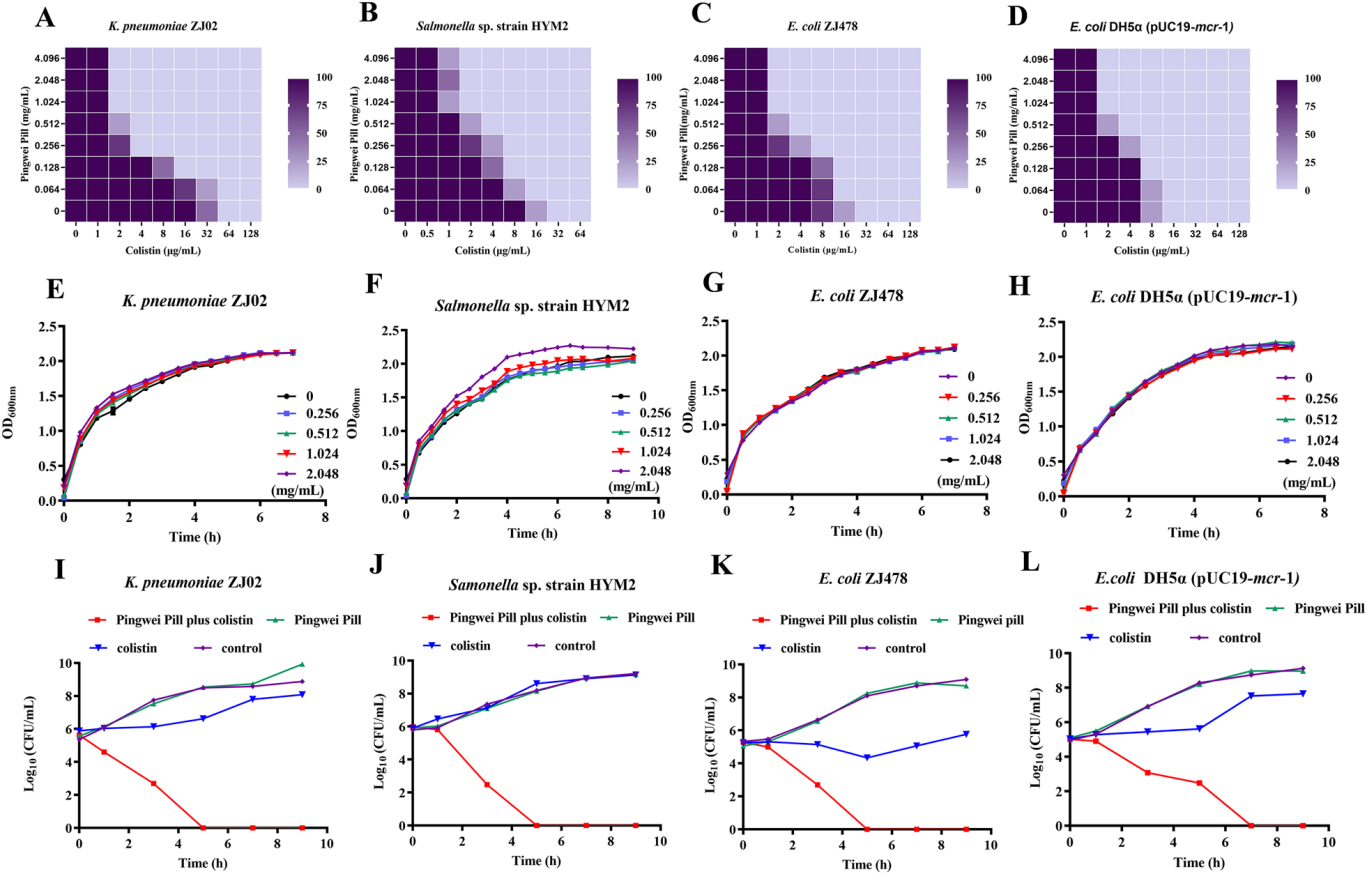
Pingwei Pill restored the activity of colistin against MCR-1-positive bacteria without affecting bacterial growth. Checkerboard dilution analysis shows the combined effect of Pingwei Pill and colistin against the *mcr*-1-positive strains *K. pneumoniae* ZJ02 (**A**) and *Salmonella *sp. strain HYM2 (**B**), *E. coli* ZJ478 (**C**) and *E. coli* DH5α (pUC19-*mcr*-1) (**D**). Color darkness or lightness represents the bacterial activity. Data were analyzed 4 times for biologically independent experiments. Growth curve of *K. pneumoniae* ZJ02 (**E**), *Salmonella* sp.strain HYM2 (**F**), *E. coli* ZJ478 (**G**) and *E. coli* DH5α (pUC19-*mcr*-1) (**H**) in the presence of the indicated concentrations of Pingwei Pill. Kill-curve kinetics of *K. pneumoniae* ZJ02 (**I**), *Salmonella* sp. strain HYM2 (**J**), *E. coli* ZJ478 (**K**) and *E. coli* DH5α (pUC19-*mcr*-1) (**L**) with the indicated treatment. Data are presented as the means ± S.D. of three biologically independent experiments

A series of antibacterial tests were performed to verify our hypothesis. Encouragingly, Pingwei Pill treatment showed a good synergistic effect with colistin against *mcr-*positive strains in vitro, with FIC indexes of 0.11 ± 0.12, 0.07 ± 0.05, 0.07 ± 0.05 and 0.07 ± 0.05 for *K. pneumoniae* ZJ02, *Salmonella* sp. strain HYM2, *E. coli* ZJ478 and *E. coli* DH5α (pUC19-*mcr*-1), respectively (Fig. 1A–D). Notably, following combination with Pingwei Pill (1.024 mg/mL), the MIC of colistin against these *mcr-*positive strains was not higher than 4 μg/mL, which suggests that Pingwei Pill treatment restored the sensitivity of colistin to sensitive states (Fig. 1A–D). However, growth curve results illustrated that the addition of Pingwei Pill did not affect the normal growth of *mcr-*positive strains (Fig. 1E–H), which indicates that Pingwei Pill treatment exerted milder selective pressure on these bacteria. Pingwei Pill combined with colistin also showed the same synergistic effect against *mcr*-3- and *mcr*-8-positive strains, with FICs less than 0.5 (Table 1). The FIC indexes were all not less than 0.5 for *mcr*-positive clinically isolated strains *K. pneumoniae* ZJ02 and *Salmonella* sp. strain HYM2 treated with Pingwei Pill and other antibiotics (such as cephalothin sodium, penicillin, meropenem, streptomycin, kanamycin, gentamycin, chloramphenicol, erythromycin, acheomycin and ciprofloxacin) (Table 1). However, a synergistic effect was not observed in the bacteria *E. coli* ATCC 25922 without *mcr* genes in the presence of Pingwei Pill in combination with colistin (Table 1). Especially, in the *Salmonella* GP9 without the gene *mcr*, however resistance to colistin, was further tested in our work. As shown in Table 1, no synergistic effect was observed for this bacterium for the combined therapy, further suggesting that Pingwei Pill is specific for the inhibition of MCR-1 activity, which specifically recovers the bacteriostasis of colistin by targeting MCR.

**Table 1:** MIC values of colistin alone or in combination with Pingwei Pill (1.024 mg/mL) in various tested strains. Data are presented as the means ± S.D (n = 3)

Species	*mcr-1* confirmation	Antibiotics	MIC (µg/mL)		
			Alone	Combination	FIC Index
*E. coli* ATCC 25,922	–	Colistin	1.50 ± 0.58	1.06 ± 0.18	1.06 ± 0.16
*K. pneumoniae* ZJ02	+	Colistin	48 ± 18.48	2.0 ± 0.00	**0.11 ± 0.12**
		Cephalothin sodium	1024.00 ± 0.00	853.33 ± 295.60	1.08 ± 0.29
		Penicillin	853.3 ± 295.60	1024 ± 0.00	1.08 ± 0.29
		meropenem	0.50 ± 0.00	0.50 ± 0.00	1.08 ± 0.29
		Streptomycin	682.67 ± 295.60	512 ± 0.00	1.25 ± 0.00
		Kanamycin	512.00 ± 0.00	426.67 ± 147.80	1.08 ± 0.29
		Gentamycin	853.33 ± 295.60	853.33 ± 295.60	1.25 ± 0.00
		Chloramphenicol	682.67 ± 295.60	682.67 ± 295.60	1.25 ± 0.00
		Erythromycin	256.00 ± 0.00	170.67 ± 73.90	0.92 ± 0.29
		Acheomycin	341.33 ± 147.80	213.33 ± 73.90	0.92 ± 0.29
		Ciprofloxacin	426.67 ± 147.80	384 ± 221.70	1.08 ± 0.29
*Salmonella* sp. strain HYM2	+	Colistin	21.33 ± 9.24	1.33 ± 0.58	**0.07 ± 0.05**
		Cephalothin sodium	42.67 ± 18.48	32.00 ± 0.00	0.84 ± 0.29
		Penicillin	21.33 ± 9.24	21.33 ± 9.24	1.00 ± 0.00
		meropenem	0.10 ± 0.04	0.08 ± 0.04	0.83 ± 0.29
		Streptomycin	341.33 ± 147.80	213.33 ± 73.90	0.72 ± 0.30
		Kanamycin	682.67 ± 295.60	426.67 ± 147.80	0.77 ± 0.31
		Gentamycin	853.33 ± 295.60	682.67 ± 295.60	1.00 ± 0.33
		Chloramphenicol	426.67 ± 147.80	256.00 ± 0.00	0.73 ± 0.29
		Erythromycin	213.33 ± 73.90	170.67 ± 73.90	0.875 ± 0.30
		Acheomycin	512 ± 0.00	341.33 ± 147.80	0.75 ± 0.32
		Ciprofloxacin	6.67 ± 2.31	5.33 ± 2.31	0.83 ± 0.29
*E. coli* DH5α (pUC19-*mcr-1*)	+	Colistin	10.67 ± 4.62	2.00 ± 0.00	**0.21 ± 0.072**
*E. coli* ZJ478	+	Colistin	13.33 ± 4.62	2.00 ± 0.00	**0.17 ± 0.07**
*E. coli* ZJ40	+	Colistin	53.33 ± 18.48	2.67 ± 1.15	**0.05 ± 0.02**
*Salmonella* GP9	–	Colistin	26.67 ± 9.24	21.33 ± 9.24	1.08 ± 0.29
*Salmonella* ZZW20 (*mcr-8* positive)	+	Colistin	42.67 ± 18.48	10.67 ± 4.62	**0.29 ± 0.19**
*Salmonella* sp. strain 15E464 (*mcr-3* positive)	+	Colistin	53.33 ± 18.48	5.33 ± 2.31	**0.10 ± 0.04**

Boldface indicates a synergistic effect with an FICI less than 0.5

**Fig. 2:**
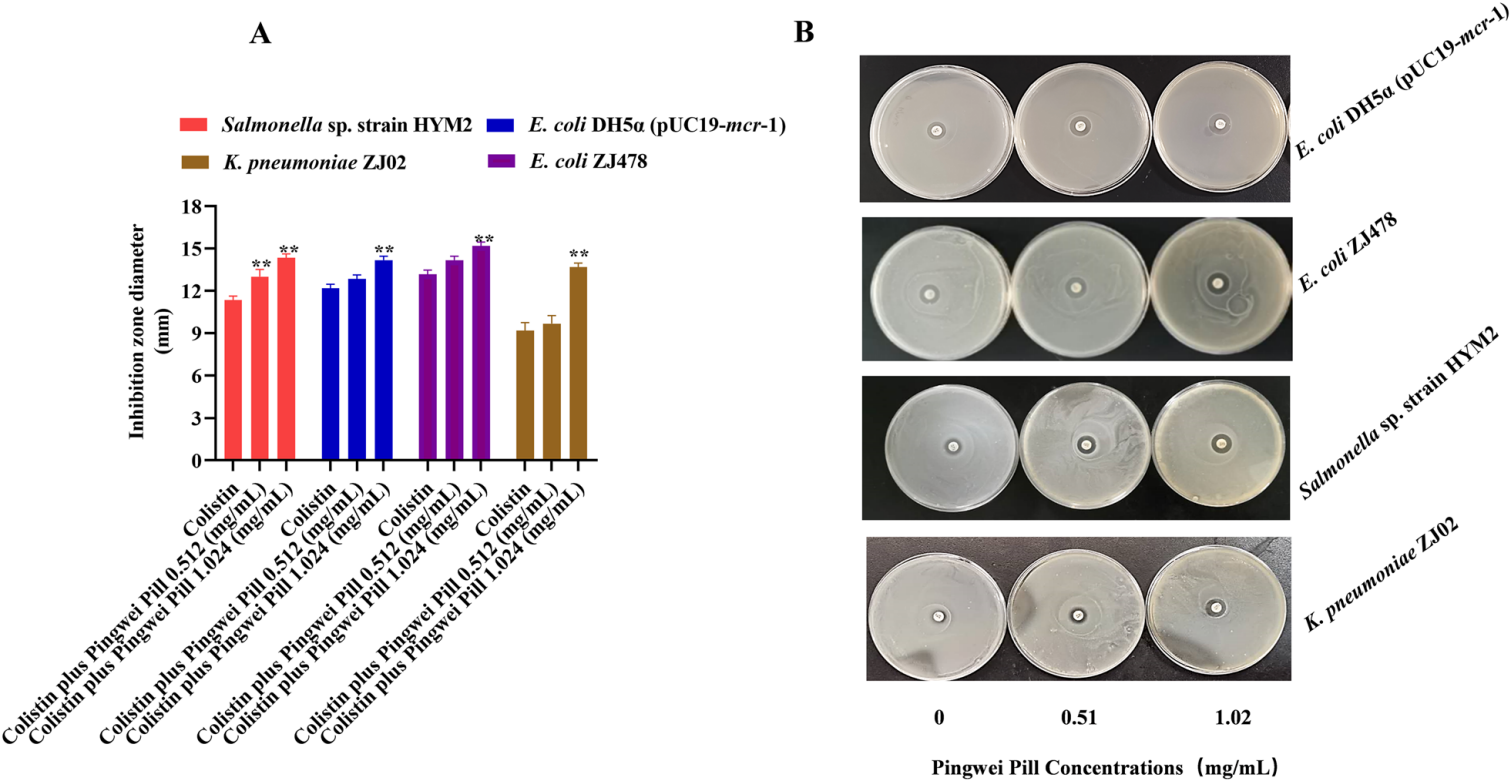
Recovery of colistin bacteriostasis against MCR-1-positive bacteria by Pingwei Pill. **A** Zones of inhibition surrounding colistin disks supplemented with 0, 0.512 or 1.024 mg/mL Pingwei Pill on lawns of *E. coli* DH5α (pUC19-mcr-1), *E. coli* ZJ478, *K. pneumoniae* ZJ02 and *Salmonella* sp. strain HYM2 on LB agar plates. Data are shown as representative pictures of each strain from one trial (n = 3). **B** Inhibition zone diameters of Pingwei Pill (0.512 mg/mL and 1.024 mg/mL) combined with colistin (10 μg) for different strains with the *mcr*-1 gene (*E. coli* DH5α (pUC19-*mcr*-1), *E. coli* ZJ478, *K. pneumoniae* ZJ02 and *Salmonella* sp. strain HYM2). Data are presented as the means ± S.D. **P < 0.01

To further validate the synergistic effects observed in the above-mentioned results, time-dependent killing curves for different growth phases of bacteria were determined. As shown in Fig. 1I–L, neither Pingwei Pill nor colistin treatment killed exponential growth or stationary phase *mcr*-positive bacteria. In contrast, the combination of 1.024 mg/mL Pingwei Pill and 2 μg/mL colistin led to visible eradication of the tested bacteria during 5–7 h, which suggests that Pingwei Pill drastically enhanced colistin bactericidal activity against MCR-positive pathogens. Consistent with these observations, the results of the CDT assay revealed that Pingwei Pill in combination with colistin significantly increased the inhibition zone diameters of MCR-positive pathogens compared to monotherapy (Fig. 2A and B). The inhibition zone diameters of *K. pneumoniae* ZJ02 expanded from 11.33 ± 0.29 mm (colistin) to 14.33 ± 0.29 mm (combination therapy) (Fig. 2B). As shown in Additional file 2: Figure S1, treatment with Pingwei Pill and colistin displayed no inhibition of MCR-1 production in the tested strains. Taken together, our results established that Pingwei Pill treatment effectively restored the antibacterial activity of colistin by specifically targeting MCR activity.

### Pingwei Pill showed a docking ability to MCR-1 without influence MCR-1 expression

**Fig. 3:**
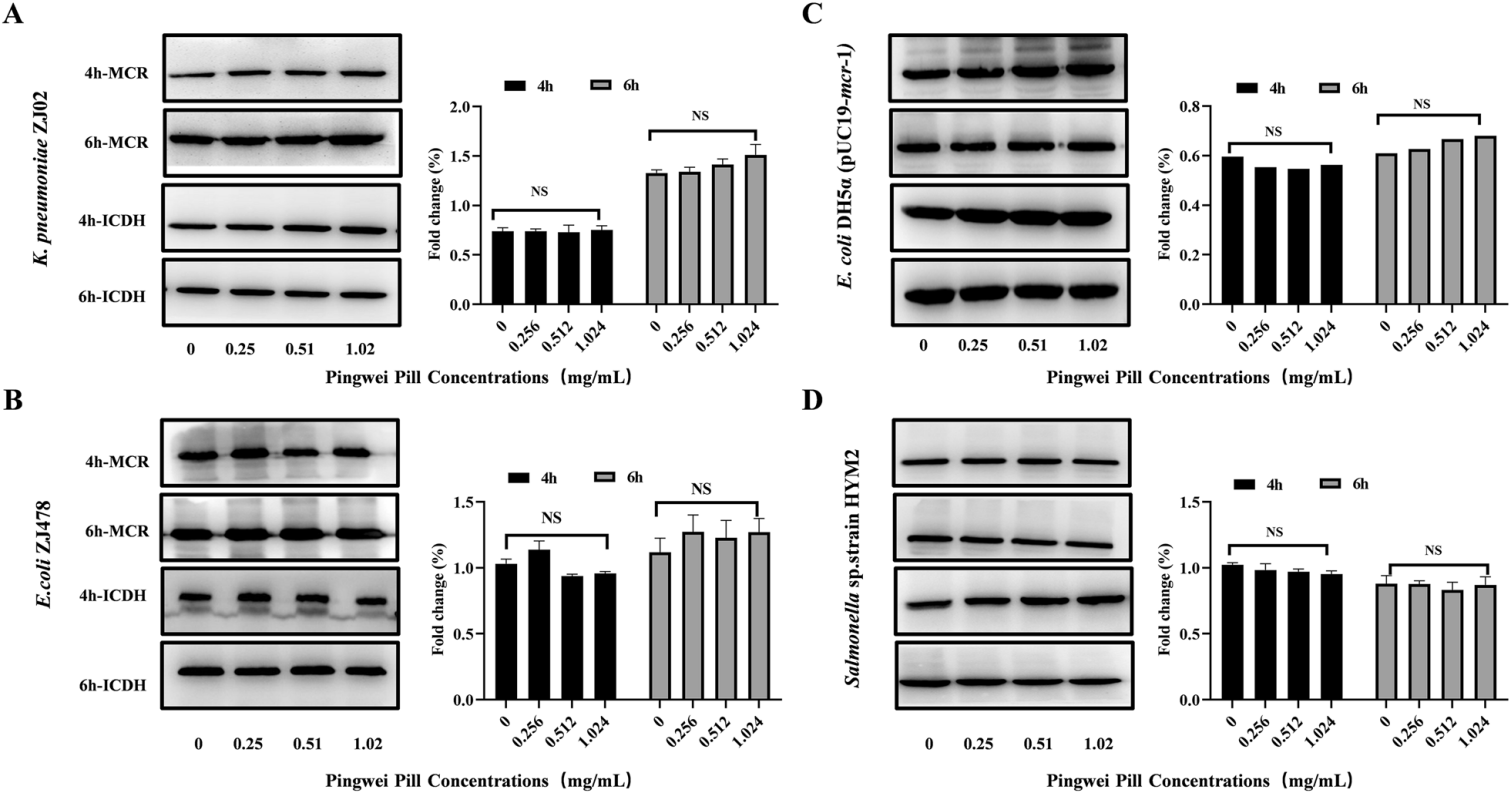
No inhibition of MCR-1 production by Pingwei Pill treatment. Bacteria carrying *mcr-*1 were cocultured with the indicated concentrations of Pingwei Pill for 4 h and 6 h. The production of MCR-1 in the cultured supernatant was determined using Western blot analysis. Blots and densitometry ratio analyses of *K. pneumoniae* ZJ02 (**A**), *E. coli* ZJ478 (**B**), *E. coli* DH5α (pUC19-*m*cr-1 (**C**) and *Salmonella* sp*.* strain HYM2 (**D**). ICDH (isocitrate dehydrogenase) as the reference. Data are shown with representative blots of each strain from one trial (n = 3)

A Western blot assay was performed to assess whether Pingwei Pill treatment affected the production of MCR in bacteria, which may contribute to the synergistic effect of Pingwei Pill with colistin. As shown in Fig. 3, Pingwei Pill treatment, at the concentrations required for the synergistic effect with colistin, produced no significant inhibition on the expression of MCR-1 when cocultured with the *mcr-1*-positive strains for 4 or 6 h, which suggests that Pingwei Pill treatment may interfere with the modification of MCR for lipid A and facilitate the membrane-damaging activity of colistin. All the research was consistent with the results of previous studies [17, 19].

**Fig. 4:**
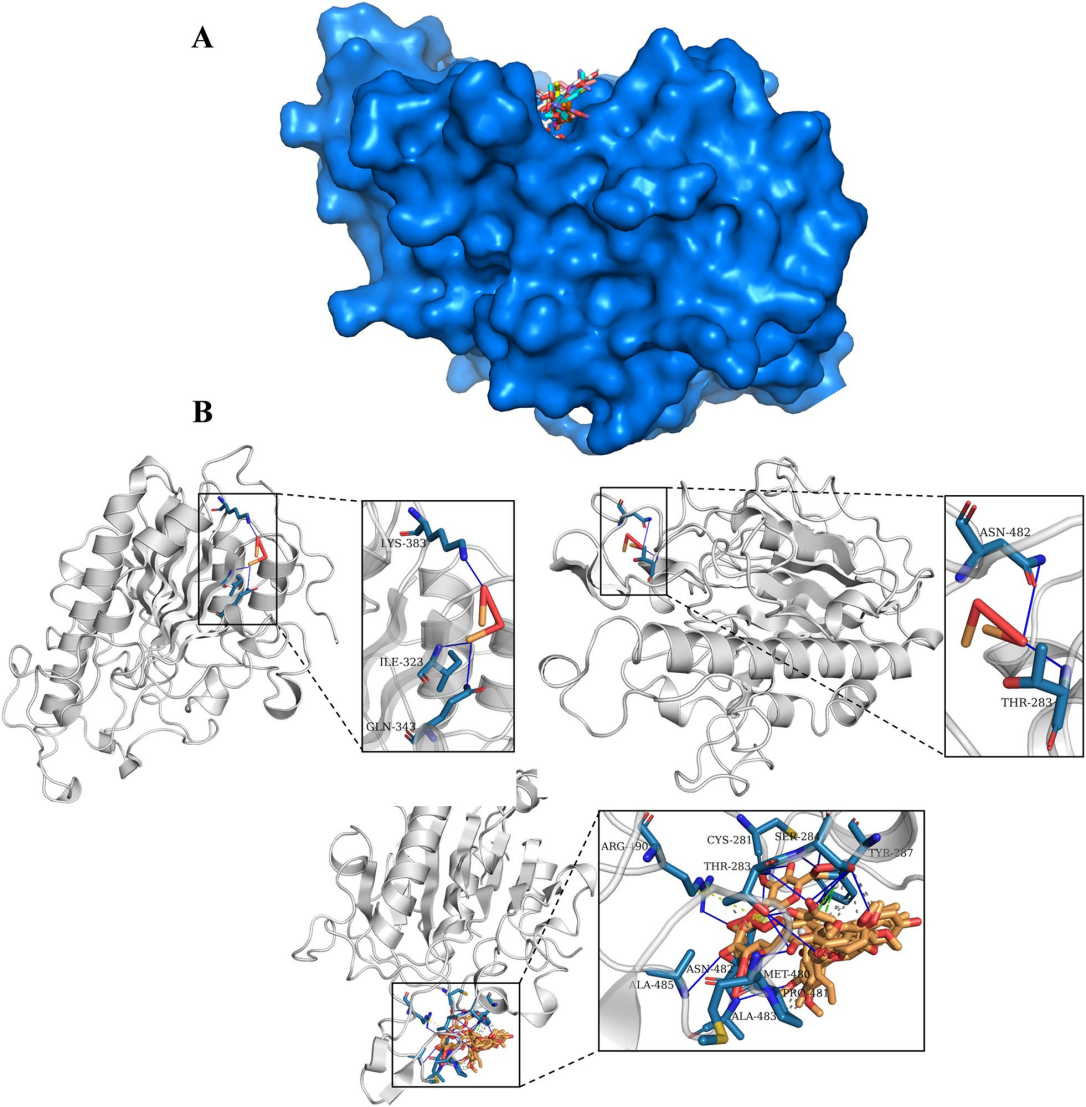
Binding sites of the crystal structure of MCR-1 (5GRR) with active compounds of Pingwei Pill. **A** is the surface binding between 5GRR and compounds. **B** is cartoon binding graph, and the left panel is the overall view, the right panel is the focused view (the 5GRR protein is gray, the binding site is gray-blue, and compounds is heavy yellow). The gray dashed line represents a hydrophobic interaction, the blue solid line represents a hydrogen bond, and the green dashed line represents π-stacking

To predict the potential activity binding to MCR-1, the main ingredients of Pingwei Pill were screened by OB > 30%, and the top7 compounds were collected to dock with MCR-1 by Sailvina software and graph visualized in PyMOL. Generally, the lower free energy indicates the more stable binding, and the chemicals with lowest gscore, binding completely in the activity pocket as shown in Fig. 4A. Specially, hydrogen bonds were formed between the top7 donors and amino acid residues at the MCR-1 active site, including SER284, CYS281, THR283, ARG490, ALA485, ASN482, MET480, TYR287. Meanwhile, the hydrophobic bonds also formed in site of THR283, TYR287, PRO 481 and ASN482 (Fig. 4B and Additional file 1: supplementary file 1, 2). Taken together, our results indicated that Pingwei Pill with multi-ingredients, can interfere with the structure of MCR and then restored the activity of colistin E.

### Pingwei Pill enhanced the membrane-damaging sensitivity of colistin without any cytotoxicity or inducing drug resistance

**Fig. 5:**
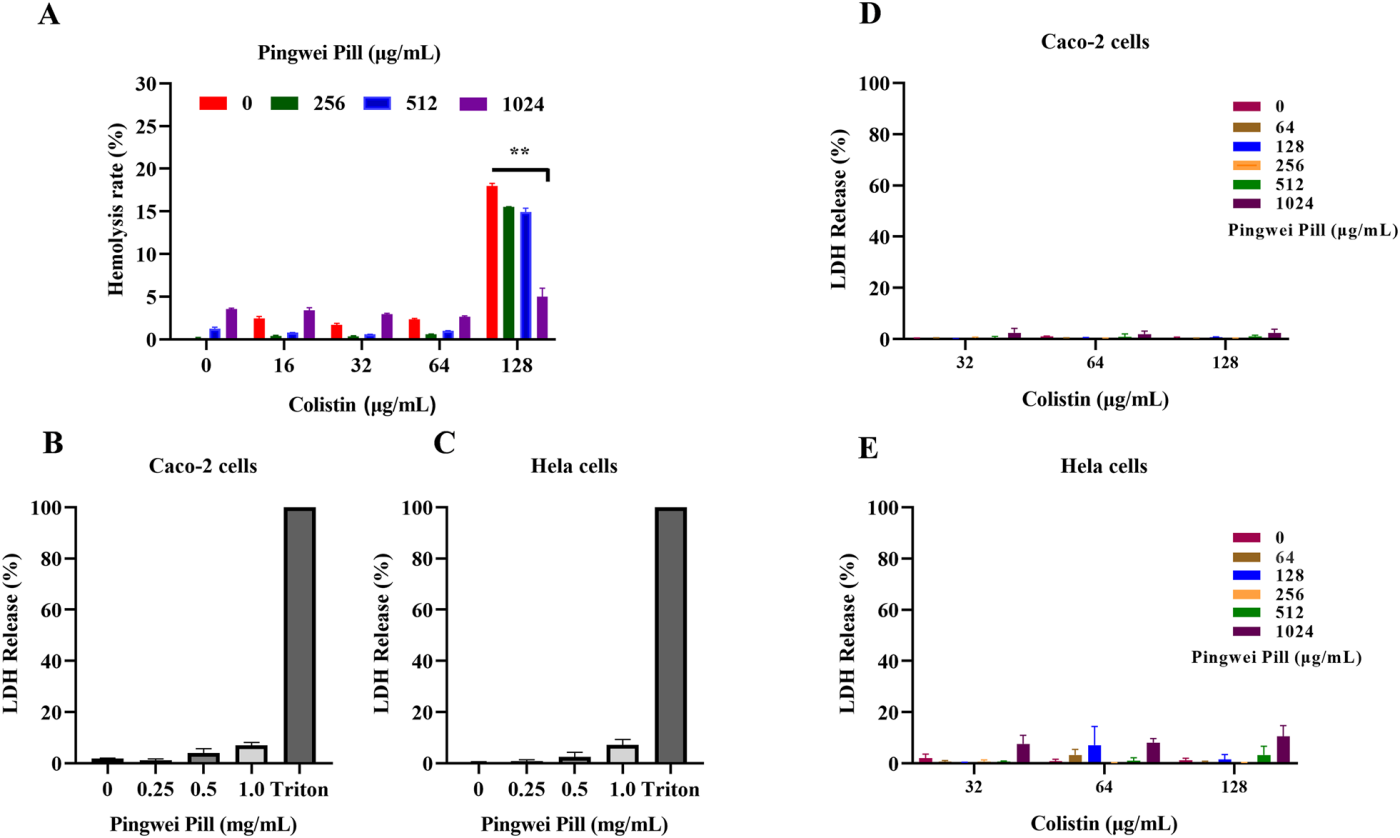
No cytotoxicity induced by Pingwei Pill treatment. **A** Hemolysis analysis of the combination of Pingwei Pill with colistin at the indicated concentrations for sheep RBCs. RBCs incubated with PBS and water were used as negative (−) and positive (+) controls, respectively. Cytotoxicity of Caco-2 cells (**B**) and HeLa cells (**C**) by determining the LDH released into supernatants following incubation of host cells with various concentrations of Pingwei Pill. Cytotoxicity of Caco-2 cells (**D**) and HeLa cells (**E**) by determining the LDH released into supernatants following incubation of host cells with the indicated treatment. NS, no significance

The concentration of Pingwei Pill (1.024 mg/mL) used for the combination therapy prompted us to further evaluate the potential cytotoxicity induced by Pingwei Pill combined with colistin. No hemolysis of sheep red cells was observed in the presence of 1.024 mg/mL Pingwei Pill. Although colistin treatment led to 16.35% hemolysis of sheep red cells, Pingwei Pill did not enhance the hemolysis of colistin but even reduced colistin-induced hemolysis (Fig. 5A). For host Caco-2 cells, HeLa cells and mouse peritoneal macrophage, Pingwei Pill (no more than 1.024 mg/mL), colistin (no more than 128 μg/mL) or combination therapy did not lead to intolerable cytotoxicity (Fig. 5B and C, Additional file 3: Figure S2A), which suggesting that the combination of Pingwei Pill and colistin for the synergistic effect against MCR-positive bacteria was relative safety in vitro (Fig. 5D and E, Additional file 3: Figure S2B). Our results indicated that Pingwei Pill, at the concentration required for the synergistic effect with colistin, did not induce cytotoxicity or exacerbate colistin cytotoxicity.

**Fig. 6:**
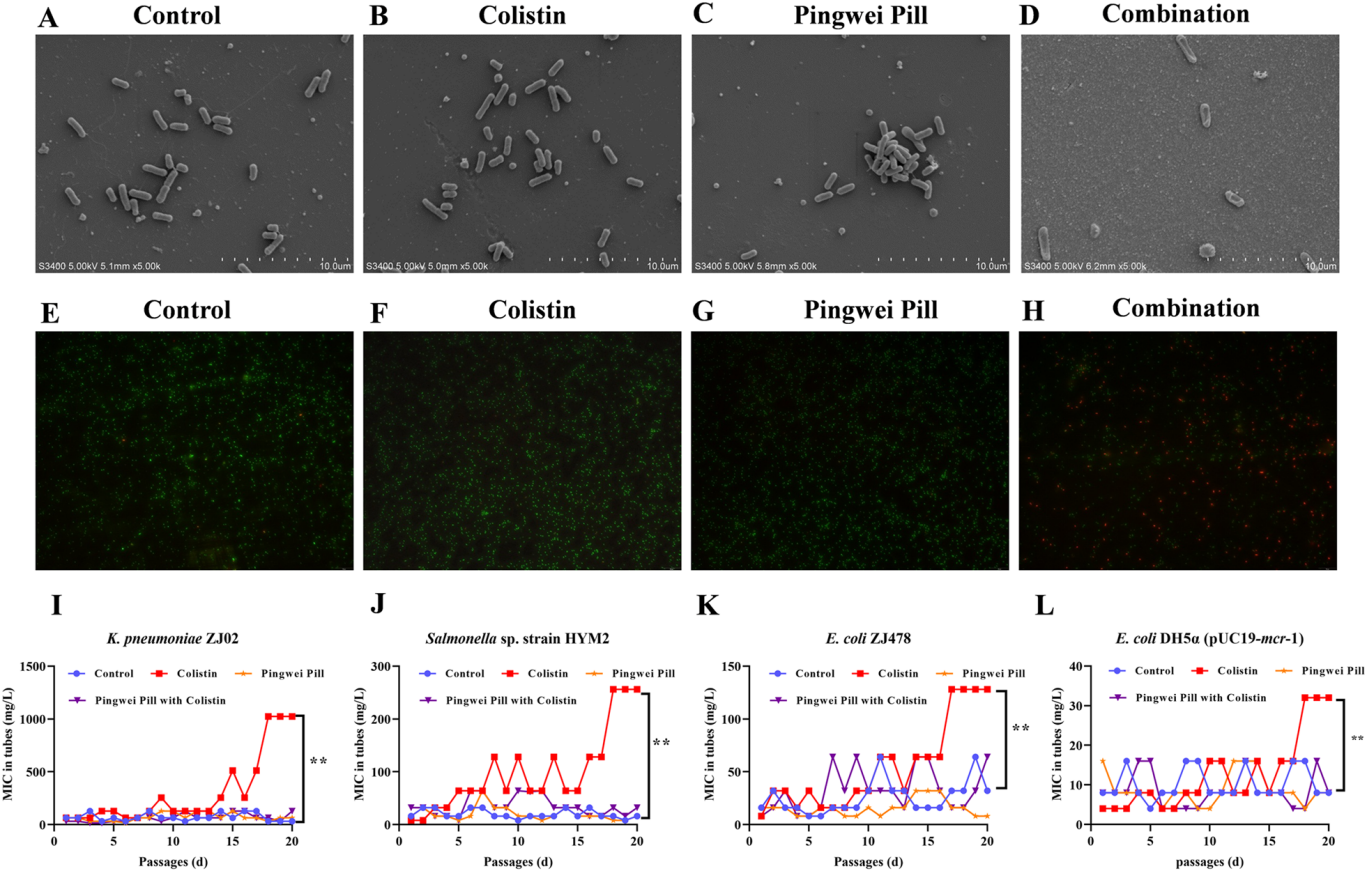
Pingwei Pill potentiated the activity of colistin in *Salmonella* sp. strain HYM2 cell membrane injury without inducing drug resistance. Following treatment with sub-MICs of colistin, Pingwei Pill or combination therapy, the morphological changes and viability of *Salmonella* sp. strain HYM2 were visualized using SEM (**A**–**D**) and a LIVE/DEAD BacLight Bacterial Viability Kit (**E**–**H**). **A**, **E** Bacteria without treatment. **B**, **F** Bacteria treated with sub-MICs of colistin (2 μg/mL). **C**, **G** Bacteria treated with Pingwei Pill (1.024 mg/mL). **D**, **H** Bacteria treated with the combination of colistin (2 μg/mL) and Pingwei Pill (1.024 mg/mL). The resistance development of various bacterial strains by serial passaging with the indicated treatment. **I** *K. pneumoniae* ZJ02 without any treatment as control, colistin (sub-MIC16μg/mL), Pingwei Pill (1.024 mg/mL), Pingwei Pill (1.024 mg/mL) plus colistin (0.5 μg/mL); **J** *Salmonella* sp*.* strain HYM2 without any treatment as control, colistin (sub-MIC 4 μg/mL), Pingwei Pill (1.024 mg/mL), Pingwei Pill (1.024 mg/mL) plus colistin (0.25 μg/mL); **K** *E. coli* ZJ478 without any treatment as control, colistin (sub-MIC 4 μg/mL), Pingwei Pill (1.024 mg/mL), Pingwei Pill (1.024 mg/mL) plus colistin (0.5 μg/mL); **L** *E. coli* DH5α (pUC19-*m*cr-1 without any treatment as control, colistin (sub-MIC 2 μg/mL), Pingwei Pill (1.024 mg/mL), Pingwei Pill (1.024 mg/mL) plus colistin (0.5 μg/mL); The y axis indicates the MIC measured directly from the tubes during the serial passages (mg/L), and the x axis is the number of passages. Colistin used as comparison was indicated. **P < 0.01

The morphological changes of *Salmonella* sp. strain HYM2 treated with sub-MICs of colistin alone, Pingwei Pill alone or their combination were investigated using SEM analysis. As expected, visible damage to the outer membrane was observed for the bacteria with combination therapy, and fewer bacteria were observed in this sample (Fig. 6A–D). The addition of combination therapy significantly increased outer membrane permeability, as evidenced by remarkable red fluorescence, and reduced green fluorescence (Fig. 6E–H). Consistent with the synergistic bactericidal activity of Pingwei Pill with colistin, much fewer bacteria were observed in the sample treated with combination therapy (Fig. 6A–H), which indicates that Pingwei Pill combined with colistin resulted in remarkable bacterial membrane injury for antibacterial activity. As shown in Fig. 6, monotherapy with Pingwei Pill or colistin did not induce membrane injury or bactericidal effects, which further suggests that Pingwei Pill treatment would not put live or dead pressure on bacteria and is consistent with the analyses of growth and time-killing curves (Fig. 1E–L). As expected, no resistance of MCR-1-positive bacteria was observed in the sample treated with Pingwei Pill for 20 passages. Conversely, the MIC values were 4–16-fold higher following 20 passages of colistin treatment for all the tested strains. Notably, like the sample treated with Pingwei Pill, the combination therapy did not induce the resistance of MCR-1-positive bacteria (Fig. 6I–L). Taken together, these results demonstrated that Pingwei Pill potentiated colistin activity by enhancing the membrane-damaging ability of colistin without inhibiting MCR production or inducing bacterial resistance.

### Pingwei Pill combined with colistin is a promising therapeutic against *Salmonella* infection in vivo

**Fig. 7:**
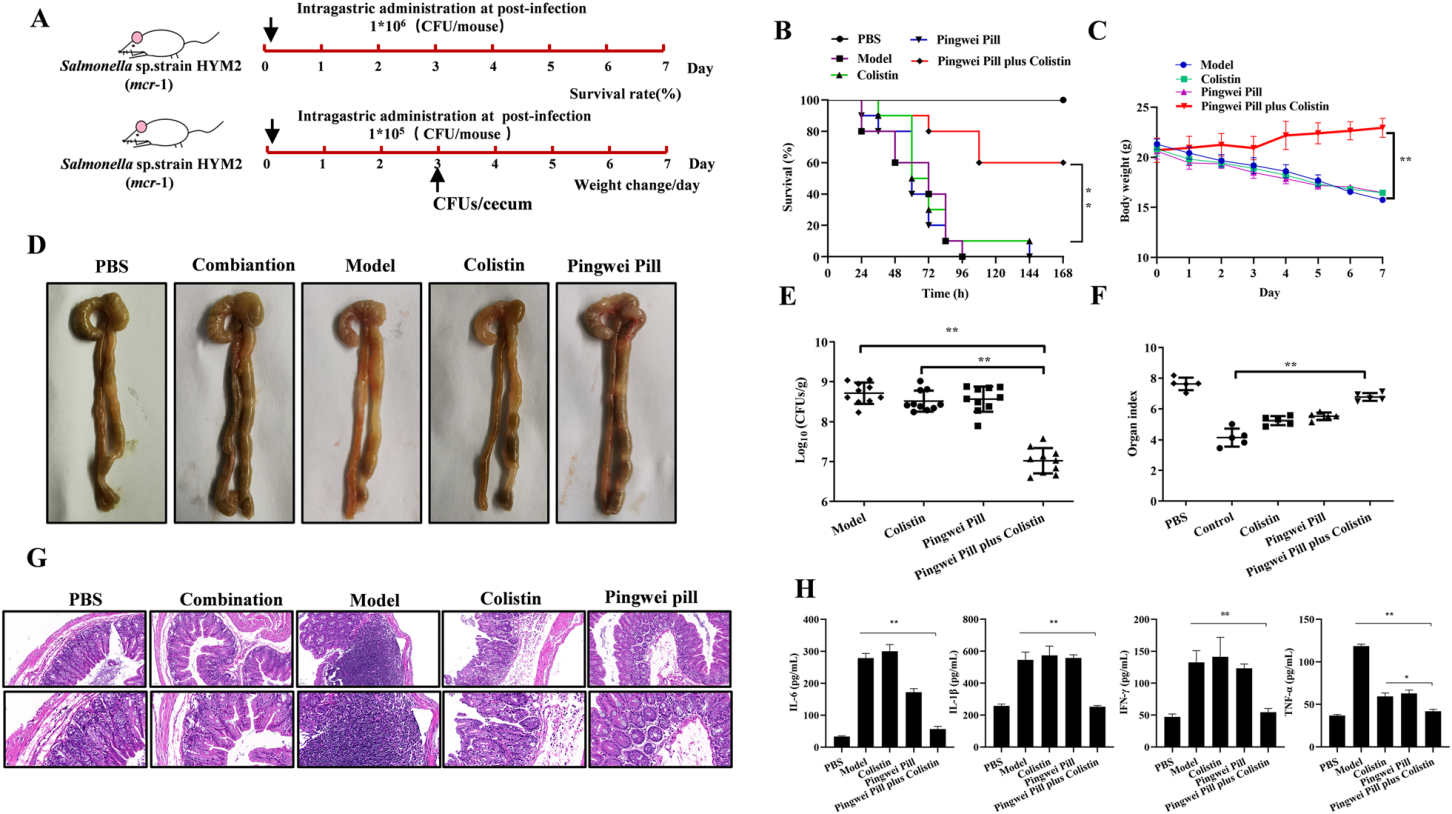
Pingwei Pill combined with colistin effectively protected mice from *Salmonella* sp. strain HYM2 infection. **A** Scheme of the experimental protocols for mouse infection. Streptomycin-treated BALB/c mice were infected with *Salmonella* sp. strain HYM2 (1.0 × 10^6^ CFUs/mouse for survival analysis and 1.0 × 10^5^ CFUs/mouse for other analyses) and treated with colistin (5 mg/kg), Pingwei Pill (0.8 g/kg), a combination of colistin (5 mg/kg) plus Pingwei Pill (0.8 g/kg) or PBS via intragastric administration. The survival (**B**, n = 10) and body weight (**C**, n = 5) of infected mice with the indicated treatment 168 h post infection. At 3 days post infection, mice (n = 10) in each group were sacrificed, and the cecum was taken for determinations of gross pathological analysis (**D**), histopathological analysis using hematoxylin and eosin (H&E) (**G**) and organ index (**F**). Following homogenization, the bacterial burden was determined by plating serial dilutions of the supernatants on agar plates (**E**), and cytokines in supernatants were examined using ELISAs (**H**). All the data are expressed as means ± S.D. (n ≥ 5). **P < 0.01

The excellent synergistic antibactericidal activity against *mcr*-positive pathogens in vitro of the combination therapy of colistin and Pingwei Pill further prompted us to verify the potential synergistic effect in vivo in *Salmonella* sp. strain HYM2-infected mouse models (Fig. 7A). Oral administration of Pingwei Pill suspended in 0.1% sodium carboxy methylcellulose (CMC-Na) with colistin was performed to prolong the therapeutic time for *Salmonella* sp. strain HYM2 infection. Following infection with lethal concentrations of HYM2, neither Pingwei Pill nor colistin rescued the infected mice. However, the combination of Pingwei Pill with colistin significantly reduced the mortality rate and bacterial colony of infected mice (Fig. 7B and E). Mice infected with sublethal concentrations of HYM2 that received the combined therapy also showed remarkably increased body weight (Fig. 7C), and organ indexes (Fig. 7F) compared to the monotherapy or without treatment. Pathological analysis revealed a visibly thin and transparent cecal wall with hemorrhagic spots, swelling and inflatable pathological changes on gross analysis (Fig. 7D). Obvious epithelial cell damage with edema and neutrophil erosion in the lamina propria and loss of goblet cells were observed on microscopic analysis for the infected mice without treatment, and monotherapy with Pingwei Pill or colistin. As expected, this pathological injury was visibly alleviated in the infected mice with combined therapy, as shown by similar observations of the ocular and microscopic morphology of the ceca in the uninfected mice (Fig. 7G). The amounts of proinflammatory cytokines, such as IL-6, IL-1β, IFN-γ and TNF-α, in the ceca were significantly higher in untreated mice infected with HYM2 than healthy mice. Following the combination treatment, the production of these cytokines was significantly decreased (Fig. 7H). Therefore, Pingwei Pill combined with colistin showed a significant protective effect in *Salmonella-*infected mice, which suggests that Pingwei Pill is a promising antibacterial synergist of colistin against MCR-1-positive bacteria.

**Fig. 8:**
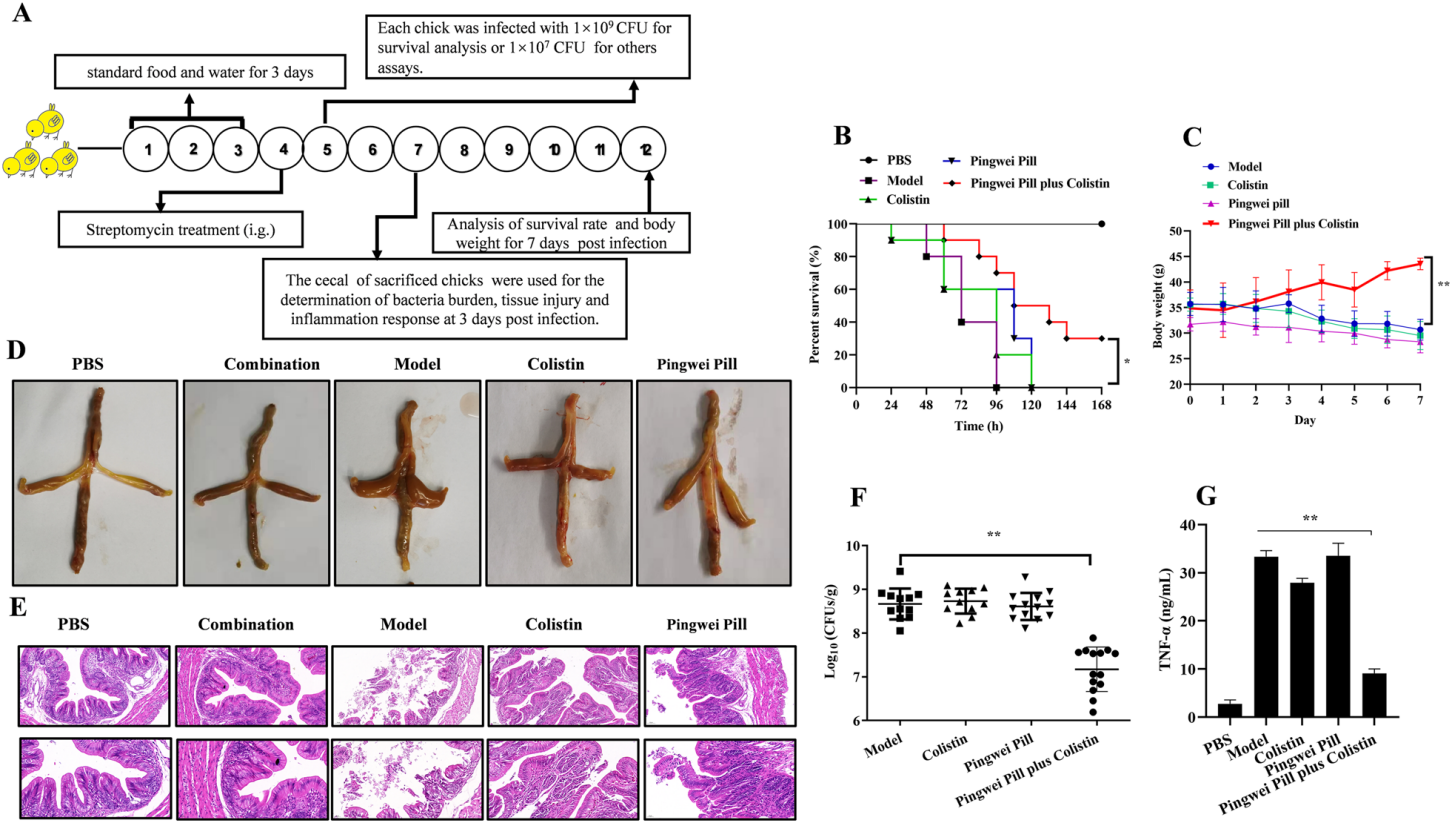
Pingwei Pill combined with colistin effectively protected chicks from *Salmonella* sp. strain HYM2 infection. **A** Scheme of the experimental protocols for the chick infection models. Streptomycin-treated one-day-old chicks were infected with *Salmonella* sp. strain HYM2 (1.0 × 10^9^ CFUs/chick for survival analysis and 1.0 × 10^7^ CFUs/chick for other analyses) and treated with colistin (5 mg/kg), Pingwei Pill (0.8 g/kg), a combination of colistin (5 mg/kg) plus Pingwei Pill (0.8 g/kg), or PBS. The survival (**B**, n = 10) and body weight (**C**, n = 5) of infected chicks with the indicated treatment at 168 h post infection. At 3 days post infection, chicks (n = 10) in each group were sacrificed, and the cecum was taken for the determination of gross pathological analysis (**D**) and histopathological analysis using hematoxylin and eosin (H&E) (**E**). Following homogenization, the bacterial burden was determined by plating serial dilutions of the supernatants on agar plates (**F**), and TNF-α in the supernatants was examined using ELISAs (**G**). All the data were expressed as means ± S.D. (n ≥ 5). **P < 0.01

The combination therapeutics of Pingwei Pill with colistin were further examined in a chick model of *Salmonella* infection (Fig. 8A). Consistent with the above results, Pingwei Pill in combination with colistin significantly prolonged the median survival time from 3 days (72 h) for infected chicks without therapy to more than 5 days (120 h) (Fig. 8B). Combined therapy remarkably increased the body weight gain by more than 10 g within 7 days compared to the infected chicks, with nearly 10 g lost to body weight (Fig. 8C). *Salmonella* infection resulted in severe swelling and local hemorrhage with epithelial cell damage and destroyed the cecal tissue structure (Fig. 8D and E). As expected, combined therapy, but not monotherapy, visibly alleviated the pathological injury (Fig. 8D and E). Pingwei Pill in combination with colistin remarkably decreased the bacterial burden (Fig. 8F) and the production of TNF-α (Fig. 8G) in the ceca compared to the samples from infected chicks that received monotherapy or PBS. Taken together, our results revealed that Pingwei Pill resuscitated the antibacterial activity and therapeutic effect of colistin against MCR-1-positive *Salmonella* infection in mice and naturally infected host chicks.

### The potential targets prediction and verification of Pingwei Pill combined with colistin

**Fig. 9:**
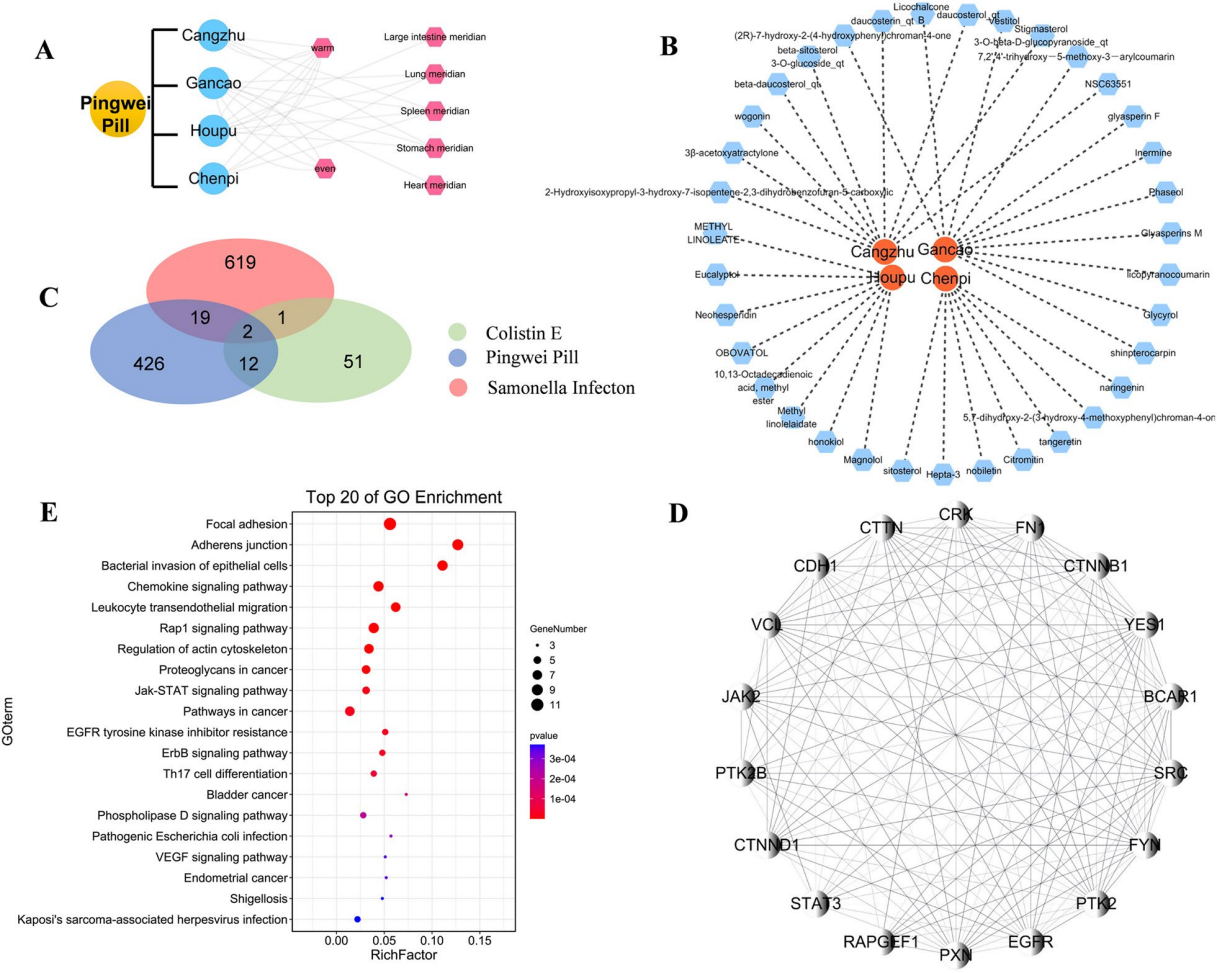
Functional enrichment analysis. **A** The formulas and herb meridian of Pingwei Pill. **B** Herbs-active ingredients network of Pingwei Pill from TCMSP database with OB > 30%. DL > 0.18. **C** Venn diagram of the potential targets of Pingwei Pill, colistin, and the therapeutic targets for *Salmonella* disease. **D** Sub-network of overlapped genes related neighbor genes was constructed using Cytoscape3.7.2. **E** GO enrichment analysis on overlapped genes and related neighbor genes

The formula of Pingwei Pill, obtained from the approved package insert, was showed in Fig. 9A, including CANGZHU, HOUPU, CHENPI and GANCAO. The four herbs of Pingwei Pill mainly belonged to the intestinal meridian, stomach meridian and spleen meridian, and treated gastrointestinal diseases. As described in above results, Pingwei Pill completely docked with MCR-1 receptor to recover the colistin activity both in vitro and in vivo, especially for the decrease of bacterial burden and cytokines level. Herein, an analysis of network pharmacology of traditional Chinese medicine was further performed to predict the potential targets of Pingwei Pill and colisitn combination therapy. All compounds of four herbs with oral bioavailability > 30% and drug-likeness > 0.18 were identified as the active ingredients, and a total of 35 active ingredients were identified based on TCMSP database (Fig. 9B and Additional file 1: supplementary file 3). Afterwards, the chemical-gene co-occurrences of colistin were collected from the PubChem database, and disease related genes of *Salmonella* infection were obtained from GEO and KEGG databases. As shown in Fig. 9C and Additional file 1: supplementary file 4, two genes, IL6 and DGKA, overlapped between the potential targets in Pingwei Pill, colistin, and the known therapeutic targets for *Salmonella* infection. IL6 and DGKA were well known to exert a vital role in cytokines mediated bacteria infection and phospholipid biosynthesis, respectively. Moreover, PPI sub-network was constructed with the hprdPPI as the background network. Then, the interaction of neighbor genes obtained based on the construction of a network screening by degree > 3 (Fig. 9D). The above key targets were input into KGEE database, and the data was download and drawn bubble graph in form OmicShare online. The key targets mainly enriched in focal adhesion pathway, adherents junction pathway, bacterial invasion of epithelial cells and chemokine signaling pathway, which consistent with results of animal test. Subsequently, all the key targets were clustered into a tree based on Kappa-statistical similarities among their gene memberships on Matescape database (Fig. 9E, Additional file 1: supplementary file 5).

**Fig. 10:**
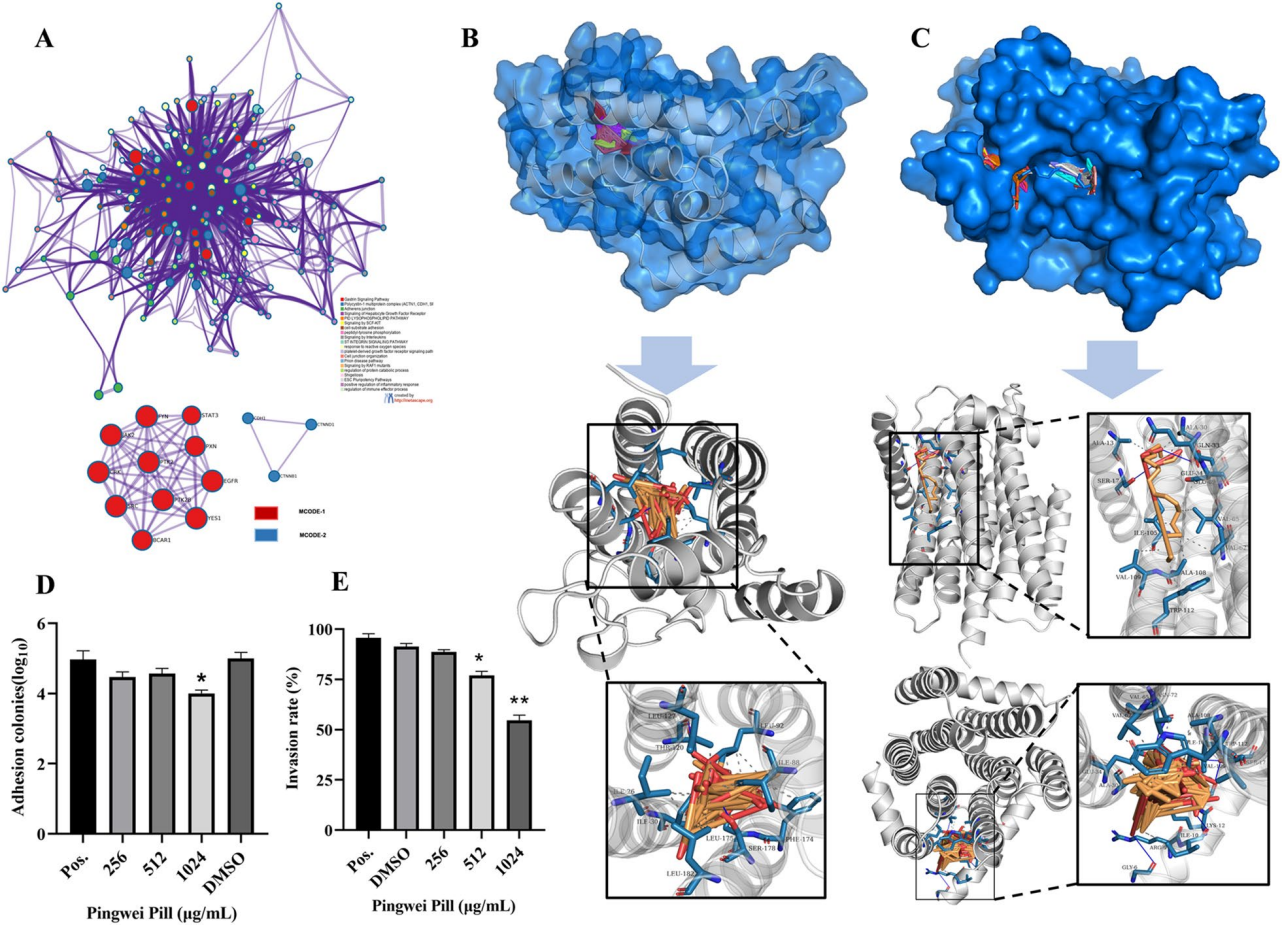
Enriched terms network, molecular docking, and pathway verification. **A** Network of enriched terms: colored by cluster ID, where nodes that share the same cluster ID are typically close to each other. Each term was represented by a circle node, where its size was proportional to the number of input genes fall into that term, and its color represent the cluster identity. Terms with a similarity score > 0.3 are linked by an edge. One term from each cluster is selected to have its term description shown as label. MCODE algorithm was then applied to this network to identify neighborhoods where proteins are densely connected. Each MCODE network is assigned a unique color. MCODE 1 was red and MCODE2 was blue. **B** Active ingredients docking with overlapped genes-IL6: blue structure was protein surface and below it: left panel is the overall view; the right panel is the focused view. **C** Active ingredients docking with overlapped genes-DGKA: blue structure was protein surface and below it: left panel is the overall view; the right panel is the focused view. **D** Bacterial adhesion assay with Pingwei Pill concentration (0 μg/mL or DMSO, 256 μg/mL, 512 μg/mL, 1024 μg/mL) combined colistin E (2 μg/mL). **E** Bacterial invasion assay with Pingwei Pill concentration (0 μg/mL or DMSO, 256 μg/mL, 512 μg/mL, 1024 μg/mL) combined colistin E (2 μg/mL)

Then, the representative terms from this cluster were selected and converted into a network layout (Fig. 10A). The network is visualized with Cytoscape (v3.1.2) with “force-directed” layout and with edge bundled for clarity. GO enrichment analysis was applied to each MCODE network to assign “meanings” to the network component (https://www.string.db.org/, http://metascape.org/). Gastrin signaling pathway of MCODE1, and polycystin-1 multiprotein complex of MCODE2 indicated that the targets of Pingwei Pill, focused on the gastrointestinal disease, and polycystin-1 multiprotein complex, played a vital role in the development regulation of cell adhesion [34, 35], which got the same perdition with KGEE database as above. Then the docking results with IL6, DGKA and top7 ingredients showed that all the active compounds were located within pocket. Hydrogen bands formed at IL6 combination site including THR-120, SER-178 and the compounds were wrapped inside the IL6 pocket, which could see only with the transparency at 60% (Fig. 10B). LEU-127, LER-92, ILE-88, ILE-182, and ILE-30 were the binding site to form hydrophobic bonds (Fig. 10B and Additional file 1: supplementary file 6). In DGKA combination site, SER-17 and GLU-69 formed hydrogen bands, ARG-9, ALA-13, GLU-34, ALA-62, TRP-112 formed hydrophobic bonds (Fig. 10C and Additional file 1: supplementary file 7). The compounds with different color could clearly observed in the bule surface of DGKA. To verificate the prediction above, bacterial adhesion and invasion assays were performed. As showed in Fig. 10D and E, Pingwei Pill 512 μg/mL plus 2 μg/mL colistin existed no inhibition to bacteria adhesion but invasion conversely. However, 1024 μg/mL Pingwei Pill plus 2 μg/mL colistin resulted in both inhibition of bacteria adhesion and invasion to HeLa cell compared with positive control without any treatment.

Taken together, our results established that Pingwei Pill is the potential candidate for *Salmonella* infection by targeting MCR-1, IL6 and DGKA genes to recover the colisitn activity, and inhibit the bacteria adhesion and invasion.

## Discussion

The time span of new drug development may be more than 10 years with unbearable cost and risk, which makes the discovery of new pharmacological effects of "conventional drugs" an alternative for bacterial infection, especially for newly emerging bacterial resistance. Unlike new drugs, the pharmacokinetics and safety of conventional drugs are more detailed, and the new use may be quickly and conveniently evaluated, which shortens the development cycle, saves development costs, and maximizes the use of resources. As is well known, medicine food homologous herbs were safety, no cytotoxicity and wide market recognition. NMPA-approved many TCM included medicine food homologous herbs just like Pingwei Pill. Therefore, new indications for conventional drugs represent an alternative avenue for drug research and development. The present study identified Pingwei Pill, an NMPA-approved TCM with a long history and wide application, as an MCR inhibitor that effectively sensitized MCR-1-positive bacteria to colistin activity with an 8- to 32-fold decrease in the MIC of colistin (FIC < 0.5). A complete bactericidal effect was observed against MCR-1-positive bacteria with the combination therapy of Pingwei Pill and colistin but not any monotherapy. Notably, at the concentrations required for synergy, Pingwei Pill treatment, unlike antibiotics, showed no influence on bacterial growth without inducing bacterial resistance. Although several natural compounds were previously reported as MCR inhibitors, the cost and risk of research and development (R&D) for these inhibitors is unbearable to obtain safety, stability, and quality control data to guarantee clinical curative effects. In contrast, Pingwei Pill has been successfully used as a compound Chinese medicinal prescriptions for spleen and stomach diseases for many years, and it was included in the 10th prepared preparations of traditional Chinese medicine issued by the Chinese Ministry of Health with widely accessible safety and effectiveness. Therefore, Pingwei Pill is an ideal potentiator with colistin without the need for a long R&D cycle and high R&D cost for clinical application.

Two animal models (mouse and chick) were used to evaluate the therapeutic efficacy of Pingwei Pill plus colistin against MCR-1-positive *Salmonella* sp. strain HYM2. Notably, the combination therapy effectively showed therapeutic efficacy in both animal model infections, with remarkably increased survival from 0% for infected animals to 80% for infected mice and 30% for infected chicks, which are one of the most important naturally infected hosts. Novel indications of conventional TCM are constantly identified, such as TCM Jianpi Wan for chronic colitis and TCM Xiaoyao Wan for chronic hepatitis. However, almost no naturally infected host infections were performed. As expected, Pingwei Pill in combination with colistin increased body weights, alleviated pathological injury and the inflammatory response in the ceca and reduced the bacterial burden in the ceca of infected animals. Superior to most other combined therapies [19, 29, 36–38], the suspension of Pingwei Pill and colistin in 0.5% CMC-Na was used for intragastric administration to fight the intestinal infection, which avoided the consideration of the C _max_ and T_max_ coincidence of these two drugs. Although 30% survival was observed for chick infection with combination therapy, the median survival prolonged from 72 h in model group to120h in combination group calculated by the GraphPad prism software 8.0.2, which further verified the efficacy of synergistic effect of Pingwei Pill with colistin.

The network pharmacology of TCM, a novel method for basic research on pharmacodynamic substances of TCM [21, 39, 40], aims to reveal the mystery of "multicomponent and multitarget" TCM prescriptions based on the system level and molecular level, which was used to further determine the mechanism of Pingwei Pill as a colistin adjuvant. All mechanistic research indicated that, the main compounds of Pingwei Pill could embed into mcr-1 active pocket, then interfered the protein conformation out of action. IL6 and DGKA were another two key targets between Pingwei Pill and colistin, the two genes were also *Salmonella* infection related genes, which mainly played a role of focal adherents, bacterial invasion of epithelial cells and chemokine signaling pathway. So Pingwei Pill might combine with colistin act on the IL6 and DGKA targets to inhibit *Salmonella* infection such as restoring the damaged intestinal epithelial surface, decrease cytokines level. All the prediction results of network were coherent with animal experiment.

## Conclusion

Pingwei Pill successfully restored the sensitivity of colistin and achieved promising clinical application value in combination with colistin for chick infection by *Salmonella* due to its potential therapeutic performance in combination therapy. Our data revealed that the combination of Pingwei Pill, an NMPA-approved TCM identified as an MCR inhibitor, and colistin represents a new therapeutic strategy for bacterial infection to meet current clinical challenges.

## Supplementary Information

**Additional file 1: Supplementary file 1.** The results of molecular docking active ingredients of Pingwei Pill docking with 5GRR, IL6 and DGKA. **Supplementary file 2.** Protein(5GRR)-Ligand Interaction Profiler. **Supplementary file 3.** Active ingredients of Pingwei Pill. **Supplementary file 4.** The potential genes or the genes overlapped of colistin, Pingwei Pill, and disease related genes of *Salmonella* infection. **Supplementary file 5.** GO Enrichment results. **Supplementary file 6.** Protein (IL-6)-Ligand Interaction Profiler. **Supplementary file 7.** Protein (DGKA)-Ligand Interaction Profiler.

**Additional file 2: Figure S1.** No inhibition of MCR production by Pingwei Pill with colistin.

**Additional file 3: Figure S2.** No cytotoxicity induced by Pingwei Pill with or without colistin treatment.

## Data Availability

Not applicable.

## Abbreviations

NMPA: National Medical Products Administration
MCR: Mobile colistin resistance
MDR: Multidrug resistance
TCM: Traditional Chinese Medicine
ATCC: American Type Culture Collection
LB: Luria–Bertani
MIC: Minimum Inhibitory Concentration
FIC: Fractional Inhibitory Concentration
PVDF: Polyvinylidene fluoride
LDH: Lactate dehydrogenase
SEM: Scanning electron microscope
THY: Todd-Hewitt-yeast
ETCM: The Encyclopedia of Traditional Chinese Medicine
TCMSP: Traditional Chinese Medicine Database and Analysis Platform
GEO: Gene Expression Omnibus
KEGG databases: Kyoto Encyclopedia of Genes and Genomes
