# Supplementary material for: NMPA-approved traditional Chinese medicine-Pingwei Pill: new indication for colistin recovery against MCR-positive bacteria infection
Source: Chin Med. 2021 Oct 18;16:106. doi: 10.1186/s13020-021-00518-y (PMC8524834; doi:10.1186/s13020-021-00518-y)
Supplement: Supplementary file 1 — Additional file 1: Supplementary file 1. The results of molecular docking active ingredients of Pingwei Pill docking with 5GRR, IL6 and DGKA. Supplementary file 2. Protein(5GRR)-Ligand Interaction Profiler. Supplementary file 3. Active ingredients of Pingwei Pill. Supplementary file 4. The potential genes or the genes overlapped of colistin, Pingwei Pill, and disease related genes of Salmonella infection. Supplementary file 5. GO Enrichment results. Supplementary file 6. Protein (IL-6)-Ligand Interaction Profiler. Supplementary file 7. Protein (DGKA)-Ligand Interaction Profiler. [file 13020_2021_518_MOESM1_ESM.pdf]

CATALOGUE

supplementary file1 -- 2

supplementary file2 -- 3

supplementary file3 -- 9

supplementary file4 -- 10

supplementary file5 -- 37

supplementary file6 -- 39

supplementary file7 -- 42

supplementary file1

The results of molecular docking: active ingredients of Pingwei Pill docking with 5GRR, IL6 and DGKA.

5GRR	Obovatol	Honokiol	Magnolol	NOBILETIN	Naringin	Eucalyptol	Citromintin
Glide gscore	-6.7	-6.7	-6.2	-3.5	147.8	-5.7	-4.7
IL6	Obovatol	Honokiol	Magnolol	NOBILETIN	Naringin	Eucalyptol	Citromintin
Glide gscore	15.5	15.7	17.8	33.2	63.5	36.4	35.3
DGKA	Obovatol	Honokiol	Magnolol	NOBILETIN	Naringin	Eucalyptol	Citromintin
Glide gscore	-8.3	-7.8	-7.7	-7.7	-11.0	-6.7	-7.7

supplementary file2

Protein-Ligand Interaction Profiler

Results

Binding Sites in DUIJIEPDB_PROTEIN

- SMALLMOLECULE
  - GOL (Glycerol)
    - GOL-A-604
    - GOL-A-605
  - UNL
    - UNL-Z-1

PLIP found one or more small problems within your structure, but was able to fix them. The analysis was finally based on this fixed structure.
Results in XML format Results in RST format
Your results will be available for 30 days using the current URL.

-
- How to Cite Us
- Run another analysis

SMALLMOLECULE

GOL (Glycerol)

GOL-A-604

Interacting chains: A

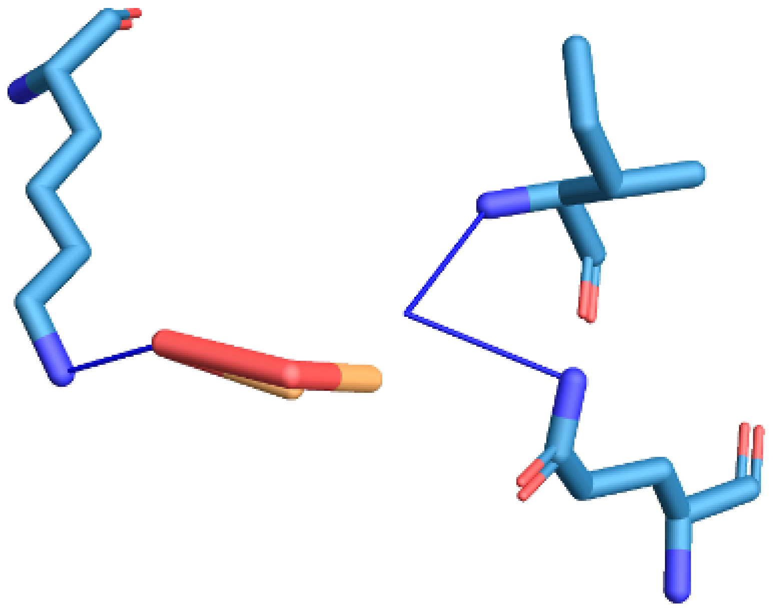

Click for 3D-View

Protein
Ligand
Water
Charge Center
Aromatic Ring Center
Metal Ion
Hydrophobic Interaction
Hydrogen Bond
Water Bridge
π-Stacking (parallel)
π-Stacking (perpendicular)

π-Cation Interaction
Halogen Bond
Salt Bridge
Metal Complexation
Download visualization in PyMol format (.pse) Download visualization as image (.png)

Hydrogen Bonds

Index	Residue	AA	Distance H-A	Distance D-A	Donor Angle	Protein donor?	Side chain	Donor Atom	Acceptor Atom
1	323A	ILE	2.25	3.18	158.55			1014 [Nam]	2765 [O3]
2	343A	GLN	2.89	3.24	102.38			1180 [Nam]	2765 [O3]
3	383A	LYS	1.97	2.93	155.86			1482 [N3+]	2761 [O3]

GOL-A-605

Interacting chains: A

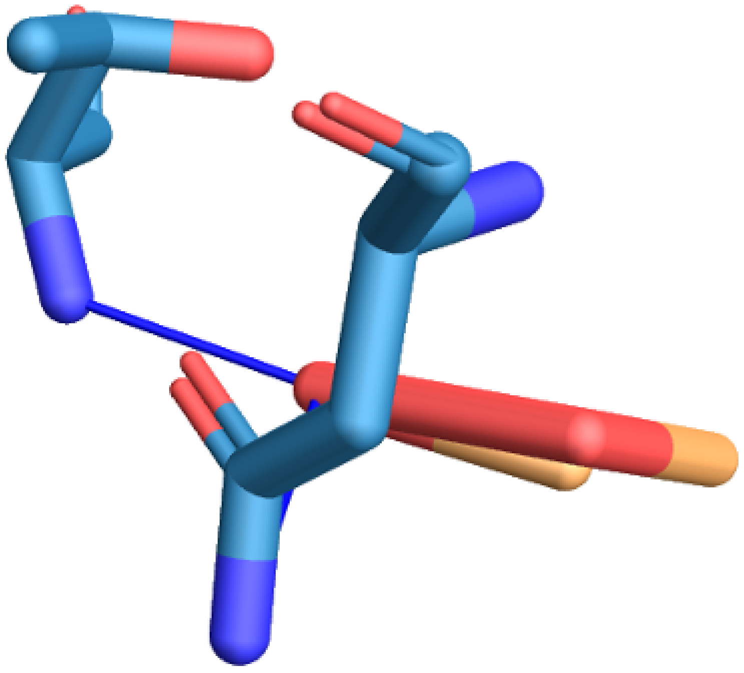

Click for 3D-View

Protein
Ligand
Water
Charge Center
Aromatic Ring Center
Metal Ion
Hydrophobic Interaction
Hydrogen Bond
Water Bridge

π-Stacking (parallel)
π-Stacking (perpendicular)
π-Cation Interaction
Halogen Bond
Salt Bridge
Metal Complexation
Download visualization in PyMol format (.pse) Download visualization as image (.png)

Hydrogen Bonds

Index	Residue	AA	Distance H-A	Distance D-A	Donor Angle	Protein donor?	Side chain	Donor Atom	Acceptor Atom
1	283A	THR	1.89	2.76	145.52			689 [Nam]	2767 [O3]
2	482A	ASN	3.36	3.98	120.87			2276 [N3]	2767 [O3]

UNL

UNL-Z-1

Interacting chains: A

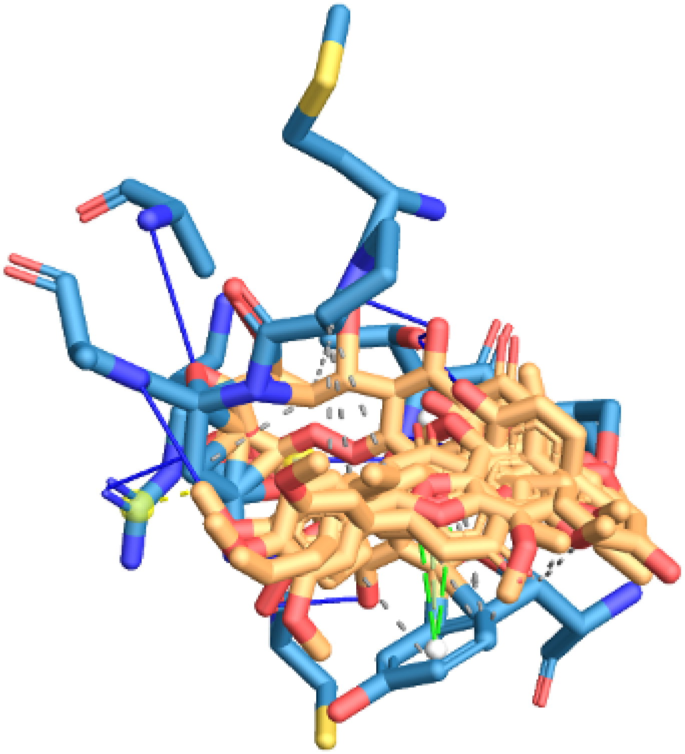

Click for 3D-View

Protein
Ligand
Water
Charge Center
Aromatic Ring Center
Metal Ion
Hydrophobic Interaction

Hydrogen Bond
Water Bridge
π-Stacking (parallel)
π-Stacking (perpendicular)
π-Cation Interaction
Halogen Bond
Salt Bridge
Metal Complexation
Download visualization in PyMol format (.pse) Download visualization as image (.png)

Hydrophobic Interactions

Index	Residue	AA	Distance	Ligand Atom	Protein Atom
1	283A	THR	3.61	94	694
2	287A	TYR	3.33	170	723
3	287A	TYR	3.55	52	721
4	287A	TYR	3.65	34	719
5	287A	TYR	3.52	30	721
6	287A	TYR	3.46	179	721
7	287A	TYR	3.60	166	718
8	287A	TYR	3.43	57	718
9	287A	TYR	3.74	106	718
10	481A	PRO	3.53	148	2267
11	481A	PRO	3.93	174	2267
12	481A	PRO	3.70	54	2267
13	482A	ASN	3.17	94	2274

Hydrogen Bonds

Index	Residue	AA	Distance H-A	Distance D-A	Donor Angle	Protein donor?	Side chain	Donor Atom	Acceptor Atom
1	281A	CYS	1.98	2.45	134.04			124 [O2]	678 [O2]
2	283A	THR	2.44	3.37	157.07			689 [Nam]	82 [O3]
3	283A	THR	3.33	3.90	154.37			81 [O3]	695 [O3]
4	283A	THR	2.15	2.53	101.47			695 [O3]	119 [O2]
5	284A	SER	2.65	3.59	159.36			696 [Nam]	120 [O3]
6	284A	SER	3.28	3.74	134.75			120 [O3]	699 [O2]
7	284A	SER	2.88	3.28	125.97			82 [O3]	701 [O3]
8	284A	SER	2.66	3.12	134.31			183 [O2]	701 [O3]
9	284A	SER	2.54	3.32	137.27			701 [O3]	29 [O3]
10	480A	MET	1.75	2.19	129.40			119 [O2]	2258 [O2]
11	481A	PRO	1.29	1.67	118.56			123 [O3]	2266 [O2]

Index	Residue	AA	Distance H-A	Distance D-A	Donor Angle	Protein donor?	Side chain	Donor Atom	Acceptor Atom
12	482A	ASN	2.07	2.57	140.26			122 [O3]	2276 [N3]
13	482A	ASN	1.86	2.35	136.32			121 [O3]	2276 [N3]
14	482A	ASN	3.07	3.86	134.61			2276 [N3]	113 [O2]
15	482A	ASN	2.91	3.14	106.00			182 [O3]	2277 [O3]
16	483A	ALA	3.23	4.01	137.30			2278 [Nam]	25 [O3]
17	485A	ALA	2.82	3.59	135.30			2294 [Nam]	125 [O3]
18	490A	ARG	2.44	2.84	103.42			2348 [Ng+]	122 [O3]

π-Stacking

Index	Residue	AA	Distance	Angle	Offset	Stacking Type	Ligand Atoms
1	287A	TYR	3.80	6.09	0.96	P	63, 64, 65, 66, 67, 68
2	287A	TYR	3.80	6.09	0.96	P	63, 64, 65, 66, 67, 68
3	287A	TYR	4.11	11.85	1.43	P	163, 164, 165, 166, 167, 168
4	287A	TYR	4.11	11.85	1.43	P	163, 164, 165, 166, 167, 168
5	287A	TYR	5.22	68.16	1.96	T	96, 97, 98, 99, 103, 104
6	287A	TYR	5.22	68.16	1.96	T	96, 97, 98, 99, 103, 104

Salt Bridges

Index	Residue	AA	Distance	Protein positive?	Ligand Group	Ligand Atoms
1	490A	ARG	4.88		Carboxylate	112, 114

Home About Help Download PLIP Privacy Legal Notice

supplementary file3

Active ingredients of Pingwei Pill

name	target
Cangzhu	NSC63551
Cangzhu	Stigmasterol 3-O-beta-D-glucopyranoside_qt
Cangzhu	daucosterol_qt
Cangzhu	daucosterin_qt
Cangzhu	beta-sitosterol 3-O-glucoside_qt
Cangzhu	beta-daucosterol_qt
Cangzhu	wogonin
Cangzhu	3β-acetoxyatractylone
Cangzhu	2-Hydroxyisoxypropyl-3-hydroxy-7-isopentene-2,3-dihydrobenzofuran-5-carboxylic
Houpu	METHYL LINOLEATE
Houpu	Eucalyptol
Houpu	Neohesperidin
Houpu	OBOVATOL
Houpu	10,13-Octadecadienoic acid, methyl ester
Houpu	Methyl linolelaidate
Houpu	honokiol
Houpu	Magnolol
Chenpi	sitosterol
Chenpi	Hepta-3
Chenpi	nobiletin
Chenpi	Citromitin
Chenpi	tangeretin
Chenpi	5,7-dihydroxy-2-(3-hydroxy-4-methoxyphenyl)chroman-4-one
Chenpi	naringenin
Gancao	shinpterocarpin
Gancao	Glycyrol
Gancao	licopyranocoumarin
Gancao	Glyasperins M
Gancao	Phaseol
Gancao	Inermine
Gancao	glyasperin F
Gancao	7,2',4'-trihydroxy − 5-methoxy-3 − arylcoumarin
Gancao	Vestitol
Gancao	Licochalcone B
Gancao	(2R)-7-hydroxy-2-(4-hydroxyphenyl)chroman-4-one

supplementary file4

Names	total	elements
ColistinE Pingwei pill Salmonella infection	2	IL6，DGKA
ColistinE Pingwei pill	12	MBL2
		TNF
		VDAC1
		SOD1
		OPRD1
		CFTR
		OPRM1
		TP53
		LYZ
		AKT1
		LCN2
		ALB
Pingwei pill Salmonella infection	19	ANXA1
		NOS2
		MAPK12
		MAPK10
		RTCB
		PPP1CC
		AURKB
		WARS
		CASP3
		DPH5
		RRM2
		MAPK3
		HIF1A
		NT5C2
		AMY2B
		TOP2B
		NFKBIA
		PYGM
		ARAF
ColistinE Salmonella infection	1	TLR4
Pingwei pill	426	ATOX1
		GAPDHS
		ITGA5
		ADRA2C
		ABCC6
		HSD17B1
		COX5A
		GLTP

		RPL19
		ATP1A3
		DCK
		COX6C
		GALK1
		MTNR1B
		PTK2B
		AADACL2
		UGT3A1
		SLCO1B3
		B3GAT1
		ALOX5
		UBA1
		R
		TRPM8
		HSD11B2
		CDK15
		ABL2
		ACVRL1
		HNMT
		PRKCB
		ABCG1
		IMPDH2
		EPHA2
		GSS
		RSL24D1
		PLK1
		GNAS
		HTR2C
		CACNB3
		GPR55
		KRTAP5-2
		CLEC4E
		LCT
		NPPB
		DRD5
		GSG2
		ALK
		TPI1
		PCMT1
		ALDH2
		YWHAE
		AFG3L2

		ORM1
		POLE3
		PNMT
		GNPDA1
		SHBG
		ABCA1
		TUBB
		MGAT1
		IGHG2
		NFKB2
		DRD3
		COX8A
		ATP5B
		GCK
		ADRA2A
		ASS1
		DAO
		ADORA2A
		EDNRA
		ABO
		B4GALT1
		ADCY5
		ESRRA
		CYP1A1
		NR0B1
		HTR3A
		CACNB1
		E
		OPRK1
		PB1
		PPARG
		GRIN2C
		BAG1
		ALOX15
		HSPA8
		NQO2
		L
		SERPINA1
		AKR1C2
		GSTP1
		PRKAA1
		GABRQ
		GABRG1

		AHCY
		MTTP
		ESRRG
		CNR1
		ABCB1
		GPR12
		AHR
		ABCC4
		CHRNA2
		ATP1A1
		PIM1
		FTL
		NR1I3
		NR3C1
		POLA1
		HCK
		CYP19A1
		SEC14L3
		TRPV2
		CDK6
		GABRB1
		PHB2
		UMPS
		LCK
		PRKAB2
		SMARCA5
		CACNA1H
		DRD2
		ABCC2
		ITPKA
		HOXA10
		AMY2A
		PPP2CB
		PRKAR1A
		HSPA2
		HSD3B1
		CSNK1G2
		GABRB2
		FHIT
		GRIN2B
		SFTPD
		APAF1
		TRPA1

		STM4066
		TNK2
		CSNK2B
		PTGS2
		KIF2C
		SULT2A1
		MT-CO3
		COX7B
		ADH1C
		GAMT
		GABRE
		TM0024
		HK1
		SIGLEC1
		ESR1
		GABRG3
		PRMT1
		TYMS
		LCTL
		GABRA2
		CALY
		COMTD1
		PDE4D
		EIF3F
		PRKAG3
		ESRRB
		ITGB2
		CACNA1F
		GRIN2A
		AK2
		PRKAG2
		GPR18
		RUVBL2
		GABRA6
		VCP
		SOAT1
		ACHE
		GSK3B
		SIGMAR1
		MTNR1A
		ADA
		POLB
		TK2

		GABRA1
		SLC2A1
		GLRA3
		LSS
		ADCY1
		TRPV1
		IGKV2-30
		HSP90AA1
		DAM
		GRIN3A
		ACSS2
		ABCC9
		VDR
		HMGCR
		AK1
		LGALS2
		DHFRL1
		NR3C2
		CACNA1C
		TK
		CACNA1D
		PRKAA2
		UCKL1
		ASNA1
		PRKAR2B
		ACSS1
		ADRBK1
		TTHA1435
		TUBA4A
		EEF2
		GLO1
		TRPM7
		HTR1A
		ATP5A1
		PPARA
		CNR2
		AMY1A
		RORA
		HTR2B
		LGALS7
		CACNB4
		MAOB
		LSM6

		ATP1A2
		NUDT9
		PPP5C
		GLRA1
		GSTM1
		COX6B1
		PYGL
		DNMT1
		HTR1B
		PLA2G1B
		CLEC14A
		ADRA2B
		HIBCH
		NAE1
		AK8
		PIK3CG
		IKBKB
		MB
		SLC25A4
		ERG11
		SIRT5
		PIK3CA
		HCN2
		GPBAR1
		ADORA1
		POLE2
		RPL15
		CREB1
		SRPK2
		TLR7
		GRIN2D
		LGALS3
		BALF5
		GRIN1
		TRDMT1
		HSPA5
		CMPK1
		SLC18A2
		DTYMK
		GNMT
		GABRD
		PAPS
		TRPV4

		GABRP
		TOP2A
		IGF1R
		TM0588
		ACVR1
		RPL3
		TAS1R2
		MTAP
		ACSL1
		ADORA3
		ABCC5
		ABL1
		ADK
		CACNA1S
		HDAC2
		GPER1
		CCT3
		ASNS
		ISG20
		HSP90B1
		NCOA1
		IGHG1
		KRTAP5-3
		FBP1
		DRD4
		EXTL2
		PI4K2B
		KHSRP
		KANSL3
		PRLR
		POLE4
		TTHA0667
		PDE10A
		CACNB2
		METAP2
		HBB
		PDE4B
		PGR
		CHRM2
		MT-CO1
		PPP2CA
		NR1H4
		RRM1

		COX7A1
		SEC14L2
		GABRA4
		COX5B
		ADORA2B
		PRMT3
		EFTUD1
		HTR1D
		POLE
		FABP6
		SIRT3
		CYP17A1
		ACTB
		RPL23
		GABRB3
		FECH
		TYR
		KIF1A
		RNASE1
		BAMF_RS28815
		ACVR1B
		GLRB
		GALE
		YARS
		GRIN3B
		RAD51
		RPL13A
		ADH1B
		AKR1C1
		DAGLA
		LPL
		JAK1
		MT-CO2
		GABRG2
		SETD7
		COX6A2
		ENPP1
		NCOA2
		GABRA5
		PIK3R1
		SEC14L4
		RRM2B
		ADCY2

		IMPDH1
		ABCB11
		RPL11
		TRPV3
		P
		CYP2B6
		RPL8
		PRKAB1
		SLC5A2
		MIF
		NFKB1
		ABCC1
		MAFF_RS13750
		ITGB3
		IL1B
		ACTA1
		WARS2
		ATP5C1
		RPL23A
		COX4I1
		HSD11B1
		RPL10L
		NCAN
		AKR1C3
		LIP3
		PFKFB4
		PTGS1
		HTR2A
		NR1I2
		GYG1
		PLA2G2E
		IFNB1
		GAPDH
		COX7C
		CEBPB
		ABCC8
		UAP1
		SULT2B1
		CDA
		EPHB2
		AR
		CALM1
		CTRB1

		HINT1
		NME1
		ESR2
		GABRA3
		RPL26L1
		CSNK2A1
		RPL37
		DAPK1
		GLT6D1
		AMHR2
		PRKCA
		SF3B3
		CES1
		STK17B
		B3GAT3
		ITGAL
		CACNA1I
		APP
		CYP1B1
		COMT
		PRKAG1
		CACNA1G
		RPS6KA3
		PNP
		CHRNA7
		ADRBK2
		UCK2
		CBR1
		SOAT2
		DRD1
		HBA1
ColistinE	51	DNASE1
		UBAC1
		NGF
		SPPL2B
		STX2
		PHF20
		ARNT
		IL24
		LIPE
		CST3
		CRP
		CCL7

		EGR1
		KLF6
		SMOX
		CCL20
		ALPPL2
		CAMP
		KCNIP1
		TPRA1
		GPX6
		WNT1
		HMOX1
		MOXD1
		STX1A
		SCGB1A1
		IL10
		CDK2AP1
		MSRB2
		ST20
		CALU
		CYP27A1
		RECK
		PCDH8
		CDAN1
		ST14
		CADM1
		MAP1LC3A
		CASP9
		CAT
		SERPINB10
		OMP
		IMPA1
		ST8SIA2
		ELL2
		BECN1
		HAMP
		DLAT
		TLR2
		GLB1
		SELENBP1
Salmonella infection	619	VAMP2
		GSKIP
		ACTR3
		GNL3

		HIVEP1
		EIF4EBP2
		CNBP
		WASF1
		ANKRA2
		KLHL2
		WASL
		HSD17B10
		HSD17B2
		CXCL3
		RAC1
		TMEM160
		ADD3
		FAM134B
		ID3
		C1QC
		MSC
		DDX11
		IZUMO4
		FAM151B
		NLRC4
		PAPOLA
		RAB3IP
		KLC4
		MSN
		MRPL38
		PTPLAD1
		EPDR1
		MRPL1
		TBC1D31
		GINS3
		CCDC34
		FGFBP3
		MRI1
		CDC25B
		CRYZL1
		CLN8
		FCGR2B
		MSL3
		VPS26A
		ROCK2
		PPP1R15B
		CCL4L2

		CD55
		CCL3L3
		PDIA3
		FOXP3
		UBE2F
		HMOX2
		BIRC5
		ZNF397
		TSPAN13
		SYT2
		DPH3
		PLEKHO1
		NCOR2
		RTF1
		CD38
		SSBP4
		DHRS7
		MSMB
		GIMAP1
		WHSC1L1
		GSTA2
		MYO6
		MINK1
		EZH2
		FBXO11
		MYO1C
		MyD88
		SWAP70
		MOSPD2
		FOXA2
		EIF1
		SLC38A10
		CDKN1B
		PDE6C
		N4BP2L2
		ACBD5
		MXD4
		PMF1
		PREP
		SPINK14
		SETDB1
		RTN3
		EIF1AX

		PCBP4
		ORC1
		MLF1
		TTLL1
		ARF4
		TAF12
		MYH9
		RHEB
		LBP
		SLCO3A1
		NCF2
		PPAP2C
		PCK2
		TF
		GUCY2C
		TLR6
		USP16
		TMEM59
		SYN1
		COMMD7
		F9
		KNSTRN
		RANBP6
		FUCA2
		CD40
		CLP1
		PIK3AP1
		TAP1
		CFH
		KDM2A
		QKI
		MRPL54
		PRAF2
		FLNC
		SPTAN1
		ARL4C
		UFL1
		HSPA4
		HPS4
		VPS41
		PIGP
		MYBL2
		ARPC3

		SMC4
		STBD1
		MGST1
		STAT5A
		MAPK9
		HGNC:9982
		CNGA1
		RNASE10
		IREB2
		SMPD2
		RBPMS
		ATP10A
		ZCCHC24
		MAGT1
		PLAGL1
		SERTAD2
		USP9X
		DYNC2H
		LMO4
		HDAC3
		HP
		BTK
		PFKL
		PFN2
		SERINC2
		COG2
		HUS1
		PRPF39
		ARPC5
		MCM3
		NUAK1
		GPBP1L1
		LSM3
		NFYC
		PGBD1
		PITPNA
		FBXW4
		MAPK14
		CCNL2
		CBLB
		SS18L2
		AKIRIN1
		FAU

		CTSS
		SNAP25
		MORC3
		IFT22
		TMEM170A
		COG4
		ALG13
		DDX26B
		MEIS1
		ZNF281
		RSRP1
		TMED7
		FST
		YPEL5
		TLK1
		MAPRE3
		SENP6
		LDLR
		FN3KRP
		MAP2K2
		CALR
		DES
		CLDND1
		IL8
		LMO2
		THY1
		MEMO1
		AP1M1
		SLC38A9
		SLC50A1
		LDHA
		OSBPL3
		JAK2
		GM2A
		DENND4B
		TEX9
		CASP1
		PPP2R5C
		PEX7
		MYH10
		TTC39C
		DNAJA1
		UBA3

		IFNGR2
		SDHD
		IMPAD1
		UBE2D3
		CPM
		TCF19
		TMEM214
		RABGAP1
		TMEM185B
		FGF12
		HNRNPK
		TLR5
		TGFBR1
		CDC42
		IMPACT
		IL18
		NCK2
		OXA1L
		SH3GLB2
		CTSK
		FLNB
		CCL4
		PDCD6IP
		EXOSC3
		FAM122A
		WAS
		SLCO1A2
		RRAGC
		AGTR1
		NDFIP2
		FAM107B
		MCM5
		RBM3
		APTX
		SNRPG
		GOLGA5
		TMEM183A
		KLC3
		RBL2
		MAT2B
		CCL4L1
		TJP1
		CD46

		AATF
		ANG
		RCN1
		TYSND1
		WSB1
		TBP
		DKC1
		GART
		NDC80
		SEC22B
		APEX1
		S100A1
		MAN2C1
		DNAJB6
		MPDU1
		ARPC1A
		RUFY1
		WDR26
		SLC37A1
		CMTM6
		PELI1
		PHYH
		POLR3C
		RAB7B
		PFN4
		CCNC
		KLF9
		TSPAN2
		MGMT
		ZFAND6
		ANKRD9
		TSTD1
		RGS18
		LFNG
		SLC25A39
		ATF4
		IFRD1
		FOXO1
		CLIC1
		ZRANB2
		TMEM41B
		FBXO7
		MAZ

		FAM26F
		ABI3
		RAP2B
		DYNC1LI
		ACADS
		RAN
		PQLC3
		UFC1
		NSDHL
		EEF1A1
		INTS3
		TOR1A
		SLC25A24
		FXR1
		BBX
		UCP2
		RAB11B
		BIN1
		PPAN
		SCLY
		DNAJB11
		PROC
		TRAPPC13
		IL1 伪
		CLK1
		CD14
		PKN2
		WDR45B
		STK24
		ROCK1
		DUSP11
		AAAS
		INPP5F
		ARPC2
		PRKCSH
		RTN4
		GGT1
		BTBD3
		GIT1
		OGT
		ABCG2
		PRR13
		GRAMD1B

		TREX2
		EPB41
		INSIG2
		NUCKS1
		TMEM158
		SERGEF
		COQ6
		RCOR1
		MYH14
		TMEM68
		MFSD7
		HTR4
		SLU7
		TRAF6
		PAPD5
		NT5C3A
		ZDHHC15
		SPAG5
		SRP14
		FAM8A1
		NAMPT
		ACOT13
		SCPEP1
		PKN3
		ACAD9
		RNASEH2C
		B3GALNT1
		CDCA3
		FOXN3
		S100A4
		FAM198B
		GPR183
		MAPK1
		KIAA0907
		TSKU
		HNRNPA2B1
		SENP2
		TMEM70
		MESDC1
		IL12RB2
		RHOU
		DCAF6
		SPTY2D1

		CXCL2
		DAZAP2
		SNAP23
		MND1
		HSPA9
		TUBGCP2
		FCHO1
		S1PR1
		FSCN1
		CAPN2
		GALK2
		CTLA4
		LEPROTL1
		AQP3
		NAA38
		SERPINB1
		NAP1L5
		FOS
		IFNG
		ABHD13
		CCDC65
		PAFAH2
		NF 魏 B1
		ENSA
		CCL3L1
		ING4
		SRSF1
		VPS28
		EEF1E1
		SZT2
		UBE2O
		ELOVL6
		MDK
		RPRD1A
		AKAP9
		PDE5A
		NCKAP1L
		MAPK8
		FEN1
		DYNC1I
		FAR1
		GNB2L1
		CALCRL

		TMBIM6
		PLAC1
		RAD54L
		AKR1B1
		TM2D2
		MPLKIP
		IER5
		MAPK13
		RBM5
		KRR1
		PRPF18
		CXCL1
		ARPC1B
		NOL6
		CHD4
		FUCA1
		MCM2
		RELA
		COMMD3
		SOCS1
		DYNC1H
		ARPC4
		OTUD5
		RAB32
		SNX3
		APOC2
		GPR56
		SDF2
		CAB39
		RHOG
		PKN1
		NFKBIB
		IDH3G
		ARFGAP3
		OGFOD1
		TMX1
		MAFB
		RILP
		DNAJB2
		LUC7L3
		MAPK11
		TMEM87A
		CA3

		TNNI1
		ATP6AP2
		ASH2L
		DCAF10
		SLC1A3
		CCL5
		SCO2
		ZFAND5
		IFNGR1
		ATP6V1G2
		MBIP
		COPB1
		PRELID1
		RABL3
		KLC1
		MLEC
		TBC1D10B
		BAG5
		AKAP13
		SDE2
		KIF3A
		PTGFRN
		CIT
		RAB11A
		CUL2
		ZNF830
		ARHGDIA
		GCAT
		TRAPPC4
		GALNT11
		ARPC5L
		FLOT1
		FAM118B
		EBNA1BP2
		NPTN
		EIF4A2
		CD47
		PPM1F
		PPM1G
		OTULIN
		PER1
		RBM39
		RAB1A

		KLC2
		YBX3
		ATG12
		RNF130
		NUDT4
		RPUSD4
		RAB9A
		SMIM15
		PFN1
		TIMM21
		PCBP2
		RHBDD3
		MAPKBP1
		SF3B4
		HIST1H2BD
		HPSE
		ZNF613
		CWC15
		PTPLAD2
		SNTB1
		APEX2
		KDELR2
		HDGF
		RAB10
		RNF114
		SFT2D1
		IFT57
		DCN
		SPAG7
		MAP4K4
		PLEKHM2
		ALKBH6
		MLH1
		SNRNP40
		HNRNPH2
		IFNAR2
		KLF10
		IL1
		PSME3
		WDR83
		COMMD6
		WNK1
		DNAJB14

		HIGD2A
		HMGA1
		GCN1L1
		CCL3
		GAS6
		ANXA4
		NDUFS3
		JUN
		MOCS2
		MTCH2
		NPL
		FBXO32
		ACTG1
		PGM3
		UBE2D2
		ATP1B3
		CENPT
		LACTB
		RNF128
		FLNA
		SRRM2
		PYCRL
		PCSK7
		DUSP9
		TMOD3
		SFRP1
		TMED4
		GATC
		AHSP
		TTC1
		HIPK1
		PYCARD
		STX4
		NEK2
		MBD1
		ACSL4
		TRAP1
		RNF44
		CMTM7
		PDLIM5
		CENPF
		WASF2
		ABHD16A

		PATL1
		SYAP1
		C9
		HDLBP
		RANGAP1
		PIGM
		RAB7A
		CSF2
		EIF2D
		EXOC6
		CTSC
		H2AFY
		PFN3

supplementary file5

GO Enrichment results

pathways in cancer	Count	Pop Hits	Pvalue
Focal adhesion	11	197	2.59E-14
Adherens junction	9	71	2.59E-14
Bacterial invasion of epithelial cells	8	72	1.32E-12
Chemokine signaling pathway	8	181	1.09E-09
Leukocyte transendothelial migration	7	112	1.70E-09
Rap1 signaling pathway	8	203	1.76E-09
Regulation of actin cytoskeleton	7	205	6.99E-08
Proteoglycans in cancer	6	195	1.64E-06
Jak-STAT signaling pathway	5	160	1.74E-05
Pathways in cancer	7	515	2.25E-05
EGFR tyrosine kinase inhibitor resistance	4	78	2.71E-05
ErbB signaling pathway	4	83	3.15E-05
Th17 cell differentiation	4	102	6.39E-05
Bladder cancer	3	41	0.00016
Phospholipase D signaling pathway	4	145	0.00021
Pathogenic Escherichia coli infection	3	53	0.00028
VEGF signaling pathway	3	59	0.00034
Endometrial cancer	3	58	0.00034
Shigellosis	3	63	0.00037
Kaposi's sarcoma-associated herpesvirus infection	4	183	0.00037
Inflammatory bowel disease (IBD)	3	62	0.00037
Prolactin signaling pathway	3	69	0.00044
GnRH signaling pathway	3	88	0.00083
Endocrine resistance	3	95	0.001
Measles	3	133	0.0024
Human papillomavirus infection	4	317	0.0024
Fluid shear stress and atherosclerosis	3	133	0.0024
Signaling pathways regulating pluripotency of stem cells	3	138	0.0025
Hepatitis B	3	142	0.0026
MicroRNAs in cancer	3	149	0.0028
Gastric cancer	3	147	0.0028
Thyroid cancer	2	37	0.0034
Axon guidance	3	173	0.004
Viral carcinogenesis	3	183	0.0045
Adipocytokine signaling pathway	2	69	0.0094
Epithelial cell signaling in Helicobacter pylori infection	2	66	0.0094
Renal cell carcinoma	2	68	0.0094
Non-small cell lung cancer	2	66	0.0094
Melanoma	2	72	0.0099

supplementary file6

Protein-Ligand Interaction Profiler

Results

Binding Sites in IL_6DUIJIE_PROTEIN

- SMALLMOLECULE
  - UNL
    - UNL-Z-1

PLIP found one or more small problems within your structure, but was able to fix them. The analysis was finally based on this fixed structure.
Results in XML format Results in RST format
Your results will be available for 30 days using the current URL.

-
- How to Cite Us
- Run another analysis

SMALLMOLECULE

UNL

UNL-Z-1

Interacting chains: A

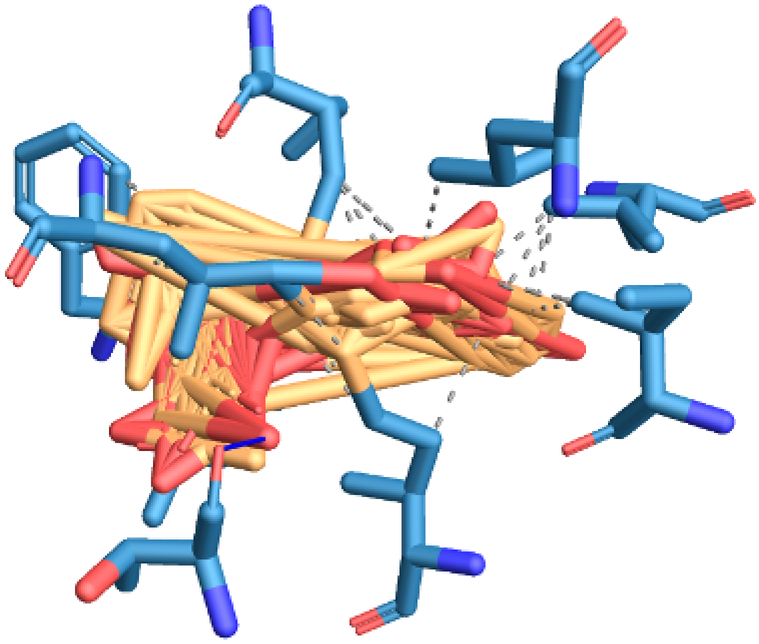

Click for 3D-View

Protein
Ligand
Water
Charge Center
Aromatic Ring Center
Metal Ion
Hydrophobic Interaction
Hydrogen Bond
Water Bridge
π-Stacking (parallel)
π-Stacking (perpendicular)
π-Cation Interaction
Halogen Bond
Salt Bridge
Metal Complexation
Download visualization in PyMol format (.pse) Download visualization as image (.png)

Hydrophobic Interactions

Index	Residue	AA	Distance	Ligand Atom	Protein Atom
1	26A	ILE	3.12	1704	68
2	26A	ILE	2.71	1730	68
3	26A	ILE	3.12	1672	68
4	30A	ILE	2.35	1776	112
5	30A	ILE	3.07	1744	112
6	30A	ILE	1.97	1719	112
7	30A	ILE	2.60	1703	112
8	30A	ILE	2.80	1808	110
9	88A	ILE	2.04	1772	666
10	88A	ILE	2.54	1699	666
11	88A	ILE	2.65	1739	666
12	88A	ILE	2.69	1729	666
13	88A	ILE	3.41	1660	666
14	92A	LEU	3.19	1708	697
15	92A	LEU	2.94	1721	697
16	92A	LEU	2.63	1719	698
17	92A	LEU	2.69	1772	698
18	92A	LEU	2.20	1703	698
19	127A	LEU	3.52	1729	1060
20	174A	PHE	3.28	1778	1528
21	174A	PHE	3.13	1723	1528
22	174A	PHE	3.20	1726	1530
23	174A	PHE	3.46	1707	1530
24	175A	LEU	2.41	1685	1541
25	175A	LEU	2.48	1775	1541
26	175A	LEU	2.88	1725	1541
27	175A	LEU	3.47	1717	1541
28	182A	LEU	3.21	1808	1609
29	182A	LEU	2.89	1705	1609
30	182A	LEU	3.43	1782	1609
31	182A	LEU	2.52	1672	1609

Hydrogen Bonds

Index	Residue	AA	Distance H-A	Distance D-A	Donor Angle	Protein donor?	Side chain	Donor Atom	Acceptor Atom
1	120A	THR	2.22	2.73	141.73			1823 [O3]	985 [O2]
2	178A	SER	2.49	3.39	156.85			1568 [O3]	1783 [O3]

Home About Help Download PLIP Privacy Legal Notice

supplementary file7

Protein-Ligand Interaction Profiler

Results

Binding Sites in TOP7DUIJIE_PROTEIN

- SMALLMOLECULE
  - OLC (Monoolein)
    - OLC-C-1120
  - UNL
    - UNL-Z-1

PLIP found one or more small problems within your structure, but was able to fix them. The analysis was finally based on this fixed structure.

Results in XML format Results in RST format
Your results will be available for 30 days using the current URL.

-
- How to Cite Us
- Run another analysis

SMALLMOLECULE

OLC (Monoolein)

OLC-C-1120

Interacting chains: B, C

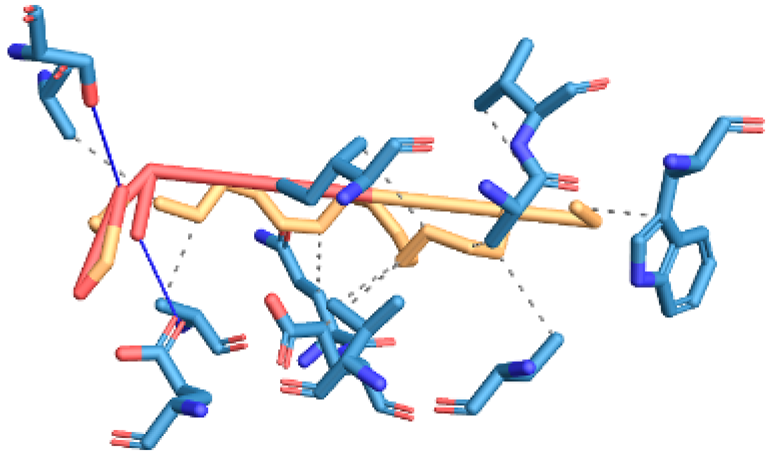

Click for 3D-View

Protein
Ligand
Water
Charge Center
Aromatic Ring Center
Metal Ion
Hydrophobic Interaction
Hydrogen Bond
Water Bridge
π-Stacking (parallel)
π-Stacking (perpendicular)
π-Cation Interaction
Halogen Bond

Salt Bridge
Metal Complexation
[Download visualization in PyMol format (.pse)] [Download visualization as image (.png)]

Hydrophobic Interactions

Index	Residue	AA	Distance	Ligand Atom	Protein Atom
1	13B	ALA	4.00	2575	949
2	30C	ALA	3.63	2576	1897
3	33C	GLN	3.74	2580	1924
4	34C	GLU	3.91	2585	1934
5	62C	VAL	3.80	2588	2138
6	65C	VAL	3.80	2585	2160
7	105C	ILE	3.85	2586	2455
8	108C	ALA	3.56	2587	2477
9	109C	VAL	3.78	2590	2484
10	112C	TRP	3.42	2591	2505

Hydrogen Bonds

Index	Residue	AA	Distance H-A	Distance D-A	Donor Angle	Protein donor?	Side chain	Donor Atom	Acceptor Atom
1	17B	SER	3.40	3.77	104.78			976 [O3]	2597 [O3]
2	69C	GLU	3.08	3.61	115.40			2594 [O3]	2192 [O3]

UNL

UNL-Z-1

Interacting chains: B, C

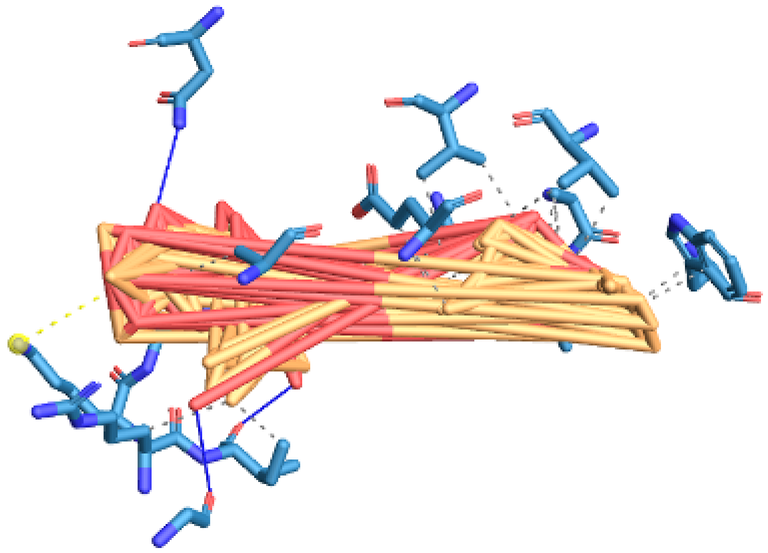

[Click for 3D-View](#)

Protein
Ligand
Water
Charge Center
Aromatic Ring Center
Metal Ion
Hydrophobic Interaction
Hydrogen Bond
Water Bridge
π-Stacking (parallel)
π-Stacking (perpendicular)
π-Cation Interaction
Halogen Bond
Salt Bridge
Metal Complexation

[Download visualization in PyMol format (.pse)](#) [Download visualization as image (.png)](#)

Hydrophobic Interactions

Index	Residue	AA	Distance	Ligand Atom	Protein Atom
1	9B	ARG	3.88	2758	913
2	10B	ILE	3.49	2758	927
3	13B	ALA	3.76	2624	949
4	30C	ALA	3.73	2624	1897
5	34C	GLU	3.73	2709	1934
6	34C	GLU	3.81	2731	1934
7	62C	VAL	3.60	2699	2138
8	62C	VAL	3.55	2697	2137

Index	Residue	AA	Distance	Ligand Atom	Protein Atom
9	65C	VAL	3.75	2614	2160
10	65C	VAL	3.86	2610	2161
11	105C	ILE	3.98	2754	2456
12	105C	ILE	3.77	2613	2455
13	105C	ILE	3.63	2614	2454
14	108C	ALA	3.78	2702	2477
15	108C	ALA	3.81	2725	2477
16	108C	ALA	3.45	2606	2477
17	109C	VAL	3.46	2724	2484
18	109C	VAL	3.48	2702	2484
19	109C	VAL	3.60	2607	2484
20	112C	TRP	3.57	2605	2505
21	112C	TRP	3.73	2728	2507

Hydrogen Bonds

Index	Residue	AA	Distance H-A	Distance D-A	Donor Angle	Protein donor?	Side chain	Donor Atom	Acceptor Atom
1	6B	GLY	3.37	3.93	152.92			2769 [O3]	890 [O2]
2	10B	ILE	2.84	3.11	109.58			2771 [O3]	923 [O2]
3	13B	ALA	2.88	3.29	127.99			2772 [O3]	948 [O2]
4	17B	SER	2.29	3.02	130.88			976 [O3]	2772 [O3]
5	17B	SER	2.19	2.72	144.14			2773 [O3]	976 [O3]
6	72C	ASN	3.32	3.95	123.93			2216 [Nam]	2645 [O3]

Salt Bridges

Index	Residue	AA	Distance	Protein positive?	Ligand Group	Ligand Atoms
1	12B	LYS	5.27		Carboxylate	2678, 2679

Home About Help Download PLIP Privacy Legal Notice
