# Supplementary figures and images for: NMPA-approved traditional Chinese medicine-Pingwei Pill: new indication for colistin recovery against MCR-positive bacteria infection
Source: Chin Med. 2021 Oct 18;16:106. doi: 10.1186/s13020-021-00518-y (PMC8524834; doi:10.1186/s13020-021-00518-y)

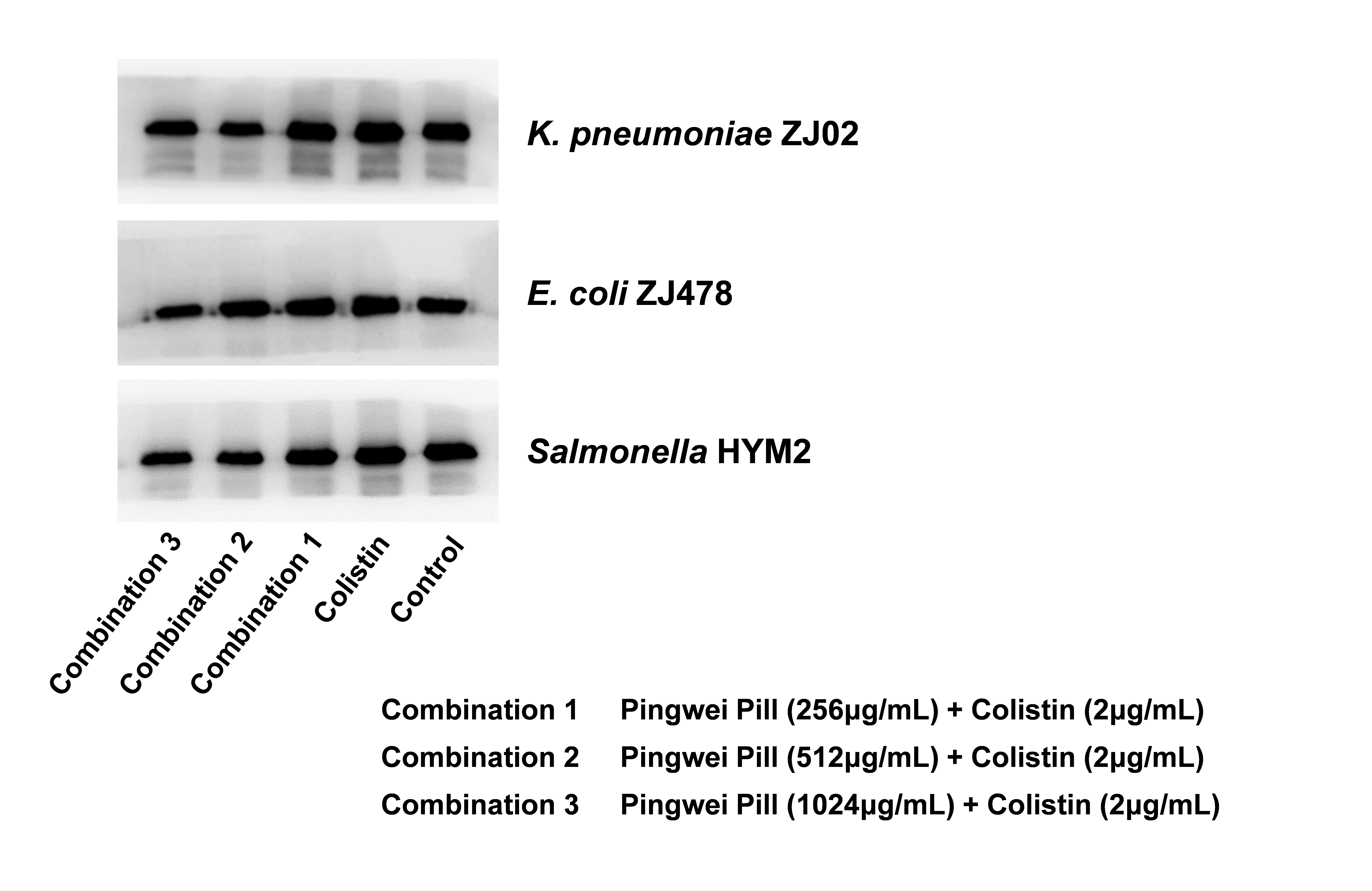

Supplement: Supplementary file 2 — Additional file 2: Figure S1. No inhibition of MCR production by Pingwei Pill with colistin. [file 13020_2021_518_MOESM2_ESM.tif]

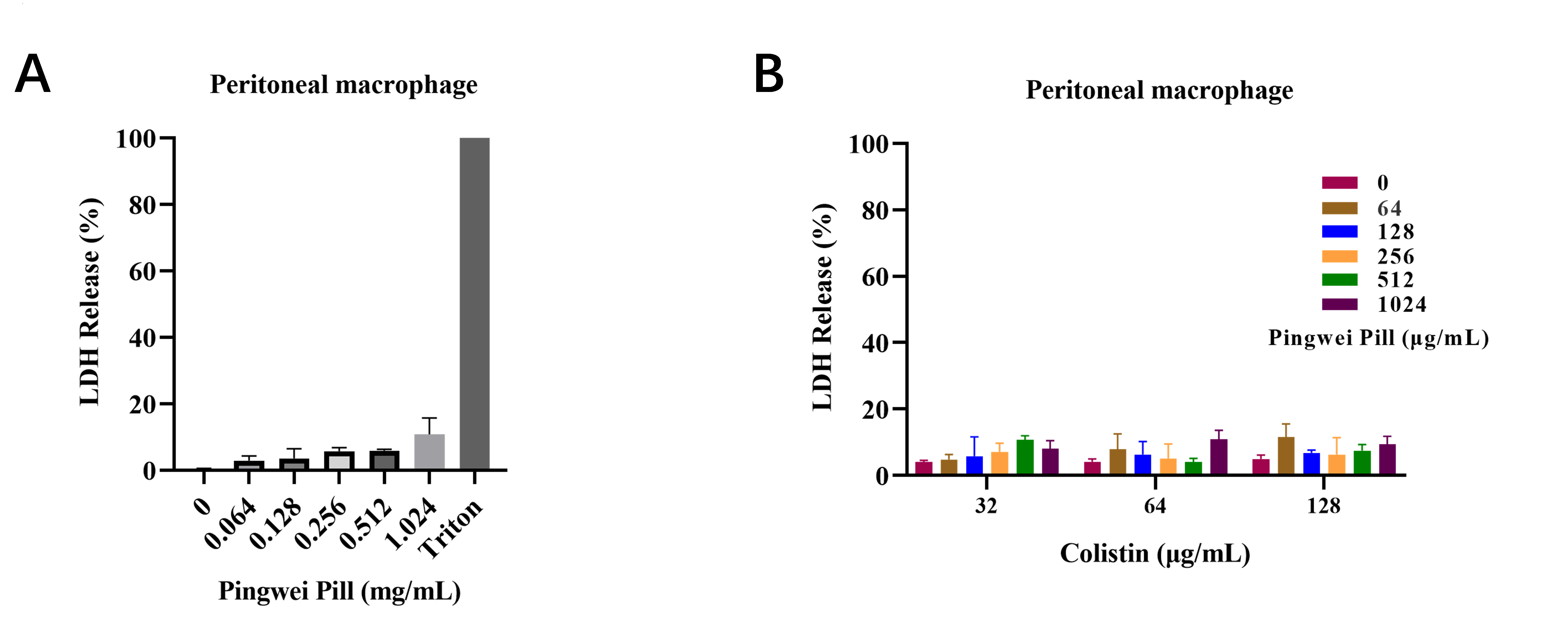

Supplement: Supplementary file 3 — Additional file 3: Figure S2. No cytotoxicity induced by Pingwei Pill with or without colistin treatment. [file 13020_2021_518_MOESM3_ESM.tif]
